# Regeneration of Planarian Auricles and Reestablishment of Chemotactic Ability

**DOI:** 10.3389/fcell.2021.777951

**Published:** 2021-11-26

**Authors:** Eugene Matthew P. Almazan, Joseph F. Ryan, Labib Rouhana

**Affiliations:** ^1^ Department of Biological Sciences, Wright State University, Dayton, OH, United States; ^2^ Whitney Laboratory of Marine Biosciences, University of Florida, St. Augustine, FL, United States; ^3^ Department of Biology, University of Florida, Gainesville, FL, United States

**Keywords:** planarian, *Girardia dorotocephala*, regeneration, stem cells, neoblast, chemotaxis, auricles

## Abstract

Detection of chemical stimuli is crucial for living systems and also contributes to quality of life in humans. Since loss of olfaction becomes more prevalent with aging, longer life expectancies have fueled interest in understanding the molecular mechanisms behind the development and maintenance of chemical sensing. Planarian flatworms possess an unsurpassed ability for stem cell-driven regeneration that allows them to restore any damaged or removed part of their bodies. This includes anteriorly-positioned lateral flaps known as auricles, which have long been thought to play a central role in chemotaxis. The contribution of auricles to the detection of positive chemical stimuli was tested in this study using *Girardia dorotocephala*, a North American planarian species known for its morphologically prominent auricles. Behavioral experiments staged under laboratory conditions revealed that removal of auricles by amputation leads to a significant decrease in the ability of planarians to find food. However, full chemotactic capacity is observed as early as 2 days post-amputation, which is days prior from restoration of auricle morphology, but correlative with accumulation of ciliated cells in the position of auricle regeneration. Planarians subjected to x-ray irradiation prior to auricle amputation were unable to restore auricle morphology, but were still able to restore chemotactic capacity. These results indicate that although regeneration of auricle morphology requires stem cells, some restoration of chemotactic ability can still be achieved in the absence of normal auricle morphology, corroborating with the initial observation that chemotactic success is reestablished 2-days post-amputation in our assays. Transcriptome profiles of excised auricles were obtained to facilitate molecular characterization of these structures, as well as the identification of genes that contribute to chemotaxis and auricle development. A significant overlap was found between genes with preferential expression in auricles of *G. dorotocephala* and genes with reduced expression upon *SoxB1* knockdown in *Schmidtea mediterranea*, suggesting that *SoxB1* has a conserved role in regulating auricle development and function. Models that distinguish between possible contributions to chemotactic behavior obtained from cellular composition, as compared to anatomical morphology of the auricles, are discussed.

## Introduction

The ability to detect external chemical stimuli is an essential tool for all living organisms. In animals, movement in response to chemical stimuli (chemotaxis) contributes to foraging and mating behaviors. In humans, chemical sensing through olfaction allows people to differentiate between pleasant odors that may be beneficial (*e.g.* nutritious food) and unpleasant odors that could be emanating from a dangerous source (*e.g.* environmental hazards and toxins). Olfaction can be lost permanently and completely (anosmia), or be suboptimal (hyposmia), due to brain trauma, aging, or congenital defects (Bromley, 2000). Olfaction can also be compromised by pathogenic infection, as seen in a significant fraction of patients affected by severe acute respiratory syndrome coronavirus 2 (SARS-CoV-2) during the coronavirus disease 2019 (COVID19) pandemic (Vaira et al., 2020). Given the association of mortality with olfactory disfunction (Pinto et al., 2014), as well as the contributions of olfaction to quality of life and diet (Reed and Knaapila, 2010), it is important to explore regenerative approaches to restore olfaction in compromised individuals.

Animals with less-developed visual capabilities, such as nematodes and mole rats, rely on chemotaxis for finding food (Ward, 1973; Catania, 2013). This is also the case for planarian flatworms, free-living members of the phylum Platyhelminthes, which not only display chemotactic behavior, but also respond to differences in temperature, contact, light, and water flow (Miyamoto and Shimozawa, 1985; Umesono et al., 2011; Inoue et al., 2015; Inoue, 2017; Ross et al., 2018). Although planarians can respond to light of different wavelengths (Paskin et al., 2014; Shettigar et al., 2017; Shettigar et al., 2021), they are not known to detect shapes (Walter, 1907). The sensory systems of planarians are well-integrated with their central nervous system (Agata et al., 1998; Okamoto et al., 2005; Inoue et al., 2015). To find food, planarians are believed to detect gradients of chemoattractants, which include amino acids leucine and tyrosine, through chemoreceptive processes modulated by calcium ion concentration (Coward and Johannes, 1969; Miyamoto and Shimozawa, 1985; Mori et al., 2019). Touch (thigmotaxis) and communication with conspecifics may also contribute to planarian foraging behaviors, but the degree by which these mechanisms are influenced by chemotaxis remains to be determined (Pearl, 1903; Iwai et al., 2010).

Anterolateral ear-like projections named auricles are believed to play a role in planarian chemotaxis (Koehler, 1932; Coward and Johannes, 1969; Farnesi and Tei, 1980; Asano et al., 1998). Electron microscopy analyses of these structures from planarians with particularly prominent auricles, such as *Dug* *esia tigrina*, identified entities that resemble chemo- and mechano-receptors embedded within ciliated epidermis of the auricle (Smales and Blankespoor, 1978; Farnesi and Tei, 1980). Recent studies have identified a handful of genes expressed in cells of the auricle, but none that display expression exclusive to these structures (Marsal et al., 2003; Nakazawa et al., 2003; Roberts-Galbraith et al., 2016; Ross et al., 2018; Auwal et al., 2020). Similarly, high throughput single-cell RNA sequencing (scRNAseq) studies failed to identify cell types exclusively present in these structures (Wurtzel et al., 2015; Molinaro and Pearson, 2016; Fincher et al., 2018; Plass et al., 2018). Therefore, it remains to be determined whether auricles serve as exclusive residence to specific sensory cells or whether their contributions to sensory functions depend on other features of their anatomy, such as position or structure.

In this study, we analyze the requirement of auricles to positive chemotactic behavior in a laboratory line of the North American planarian *Girardia dorotocephala*. Using an assay based on scoring feeding success, we found that amputation of auricles largely reduces positive chemotactic behavior. This loss is observed 1-day post-amputation (1 DPA) and restored by the second day post-amputation, which is days prior to visible regeneration of original auricle morphology. Irradiation prior to amputation abolished auricle regeneration, but did not abolish restoration of some chemotactic capacity 2 DPA. Characterization of differential gene expression in auricle tissue by high-throughput RNA-sequencing (RNA-seq), as well as a corresponding list of genes of interest with enriched expression in the auricle, are included as part of this study. This work represents an advancement in our understanding of auricle function and regeneration, while also providing a system for future studies of stem-cell mediated restoration of sensory neurons and auricle development*.*

## Materials and Methods

### Animal Husbandry

Planarians purchased from Carolina Biological Supply Company (Item #132970; Burlington, NC) were used to generate a clonal line characterized as asexual *Girardia dorotocephala* MA-C2 (Almazan et al., 2018) as well as a mixed non-clonal population (coined “wild”) of asexual *G. dorotocephala*. This clonal line was used in most experiments, but non-clonal lines were also used in structural analyses of auricle regeneration. *G. dorotocephala* cultures were maintained at room temperature in plastic containers filled with approximately 1 L of 0.75× Montjüic salts (Cebria and Newmark, 2005) in dark incubators, but with natural illumination from a laboratory window at least 10 feet away, as well as irregular short exposures to artificial light. Colonies were expanded by natural fission, as well as through amputation when increased expansion to establish a clonal line was needed. For feeding, planarians were placed on a benchtop and fed chunks of Golden Forest organic calf liver (Fremont Beef Company, Fremont, NE) at room temperature once or twice per week. The liver was purchased frozen, cut into single serving pieces or pulped, stored at −80°C in aluminum foil or in small plastic Petri dishes (respectively), and thawed before use. Planarians were not fed during the week prior to analysis or experimental procedures.

### Chemotaxis Assays

Positive chemotactic behavior was assessed using *G. dorotocephala* of 1.0–1.5 cm in length. A blue 5.7 L (6 qt) container (Sterilite^®^, Townsend, MA) measuring 35.6 × 20.3 × 12.4 cm (14″ L × 8″ W × 4 7/8″ H) was used as a feeding arena. The container was filled with 1.5 L of 0.75× Montjüic salts and a sterile 35 mm petri dish (Falcon^®^, Tewksbury, MA) was placed in the middle of the container as a feeding pedestal 1 cm from the bottom of the container. 40 µl pellets made from a mixture of 500 µl of liver puree, 200 µl of 2% TopVision Low Melting Point Agarose (Thermo Scientific, Waltham, MA; dissolved in ultrapure water), and 7 µl of Assorted Food Color & Egg Dye (McCormick & Company, Inc, Hunt Valley, MD), were used as chemoattractant. Pellets were prepared within 24 h of experimentation and stored at 4°C before use. Planarians were placed in the arena and allowed to habituate for 3 min before placing three feeding pellets in the center of the pedestal. At this point, planarians were monitored for 30 min and scored every 3 min based on observation of active feeding or detection of food dye in the gut of individual planarians.

An assay to assess the vertical distance range that elicits a chemotactic response was performed in 4 L polypropylene graduated cylinders (Nalgene, Rochester, NY) filled with 3.75 L of 0.75× Montjüic salts, with the chemoattractant placed inside a perforated 5 ml microcentrifuge tube (Phenix Research Products, Candler, NC) suspended at varying heights with a fishing line. For this assay, planarians were positioned in the bottom of the graduated cylinder and allowed to habituate for 3 min, the feeding pellets were then placed inside the perforated 5 ml microcentrifuge tube and positioned at 5, 10, and 45 cm from the bottom of the graduated cylinder, at which point feeding was scored in 10 min intervals for a total of 4 h.

All chemotaxis assays were performed with the laboratory room lights turned off and with 2 ft-tall cardboard surrounding the feeding arenas to decrease the natural light that came from laboratory windows. These experiments were run with groups of 8–13 planarians, and the data from a minimum of three independent analyses were used to calculate means and statistical significance using unpaired two-tailed Student’s *t*-tests.

### Manipulation of Planarians Prior to Chemotaxis Assay

Amputation of auricles and other head fragments were performed under a dissecting microscope by immobilizing planarians on a 2-fold ply of Whatman filter paper #1 (Whatman Paper Limited, Kent, England) dampened with 0.75× Montjüic salts and placed on an aluminum block pre-cooled on ice. After amputations were performed using a size 11 disposable scalpel (EXELINT International Corporation, Redondo Beach, CA), planarians were placed back in standard husbandry conditions until the day of the analysis with at least one water change after amputation.

To analyze the contribution of neoblasts to chemotactic behavior and auricle regeneration, planarians were then subjected to 15 min treatments with 110 kVp in a Faxitron X-ray irradiation cabinet [Model 43855A (110 kVp, 3 mA), Faxitron Bioptics LLC, Tucson, AZ] as per Tasaki et al. (2016). Amputation of auricles was performed 3 days-post irradiation and followed by chemotaxis assays 1, 4, 7, and 11 days post-amputation.

### Immunofluorescence

Planarians were fixed for immunofluorescence using two different approaches. For initial analyses of mitotic cells and the nervous system, fixation was carried out as described by Forsthoefel et al. (2014) with slight modifications. Briefly, planarians were sacrificed by incubating for 6 min in 2% HCl, followed by incubation in Methacarn Solution (6:3:1 methanol:chloroform:acetic acid) for 20 min at room temperature with slow nutation on a rocking platform. Samples were then incubated in PBSTx (PBS supplemented with 0.3% Triton-X), 1:1 PBSTx:methanol, 100% methanol, and then bleached under white light in methanol containing 6% hydrogen peroxide. For analysis of ciliated structures and detailed timepoints of regeneration after auricle amputation, fixation took place as per Ross et al. (2015). Planarians were sacrificed by incubating for 8 min in cold 2% HCl ultrapure water solution on a rocking platform and fixed in a solution of 4% formaldehyde in PBSTx for 1 h at 4°C. After fixation, samples were rinsed in PBSTx and bleached in PBSTx supplemented with 6% hydrogen peroxide overnight at room temperature under a white light. Samples were then rinsed with PBSTx, incubated for 2 h at room temperature in a blocking solution composed of PBSTx supplemented with 0.6% Bovine Serum Albumin (Item No. A7906, Sigma-Aldrich, St. Louis, MO) and 0.45% Fish Gelatin (Item No. G7765, Sigma-Aldrich, St. Louis, MO), and incubated overnight at 4°C with blocking solution supplemented with anti-synapsin (SYN; anti-SYNORF1; 1:250 dilution; clone ID: 3C11, Developmental Studies Hybridoma Bank, Iowa City, IA), anti-acetylated alpha-Tubulin (AcTub; 1:100 dilution; clone: 6-11B-1, Sigma-Aldrich, St. Louis, MO), and/or anti-histone H3 phospho-Ser10 (PH3; 1:250 dilution; Item no. 44-1190G, Invitrogen, Carlsbad, CA). Samples were washed in PBSTx four times for at least 15 min each at room temperature, incubated in blocking solution supplemented with Alexa Fluor^®^ 488 and/or Alexa Fluor^®^ 568 secondary antibodies (1:500 dilution; Catalog No. A-11001 and A-11011, respectively, ThermoFisher, Waltham, MA) for 3 hours, and washed four more times in PBSTx prior to mounting in a 4:1 glycerol:PBS solution. 4′,6-diamidino-2-phenylindole (DAPI) was added during incubation with secondary antibodies to visualize cell nuclei (1 μg/ml, final concentration; Item No. 28718-90-3, ACROS Organics, Fair Lawn, NJ), and FITC-conjugated Concanavalin A (Con A; 1:1,000 dilution; Vector Laboratories; Burlingame, CA) was included during secondary antibody incubation when staining epidermal cell junctions as per Zayas et al. (2010).

### Differential Expression Analysis by RNAseq

Details of analyses in this section including command lines, scripts, and data files are available online: https://github.com/josephryan/Almazan_et_al_auricles_regen

Paired-end Illumina HiSeq^®^ 2500 Sequencing System reads from this study and a previous study (Almazan et al., 2018) were used for RNAseq analyses. Reads are deposited under National Center for Biotechnology Information (NCBI) BioProject I.D. PRJNA317859 and NCBI Accession No. SRX1744820 – SRX1744825.

We used Trinity version 2.12.0 (Haas et al., 2013) to generate a reference transcriptome by concatenating RNA-Seq data from auricle fragments (SRR3479048) and from intact individuals (SRR3479052) from the MA-C2 *G. dorotocephala* clonal line (Almazan et al., 2018). We used the ‘--include_supertranscripts’ option to generate SuperTranscripts (where unique and common sequence regions among splicing isoforms are collapsed into a single linear sequence), which were used as reference transcriptome for downstream analyses. The resulting assembly is available here: https://corescholar.libraries.wright.edu/biology/802/. This new transcriptome is composed of 268,178 contigs.

We tested for the presence of contamination in the assembled transcripts by using alien_index version 3.00 (Ryan, 2014). The alien_index analysis included BLAST searches for each *G. dorotocephala* transcript against a database that included gene sets of 22 Platyhelminthes species from Wormbase Parasite, 12 non-Platyhelminthes animal species, five non-metazon eukaryotic species, five Bacteria species, and two Archaea species. The alien_index program takes that BLAST report and generates information about potential contaminants by looking specifically for instances where the best BLAST hit does not come from one of the 22 Platyhelminthes datasets. We found 2.3% of transcripts had a better BLAST hit to a non-Platyhelminthes sequence and less than 0.4% had alien_index indices greater than the standard cutoff of 40 (indicative of contamination or horizontal gene transfer).

We compared relative differences in gene expression between *G. dorotocephala* auricles (one group of auricles removed from MA-C2 and two groups of auricles removed from non-clonal cultures) and bodies (a group of intact MA-C2, a group of intact bodies from non-clonal culture, and a group of bodies from non-clonal culture post-auricle amputation) by mapping paired reads from each group to the new reference transcriptome using the CLC Genomic Workbench RNAseq Analysis platform (default settings; QIAGEN, Hilden, Germany). Genes (i.e. supercontigs) represented by less than 0.1 cumulative TPM across samples were removed from differential expression analyses. Illumina paired reads from body groups and auricle groups were mapped to the reference transcriptome (104,470,274 to 154,327,684 input reads/group) with over 93% mapping efficiency. Principal Component analysis of mapped reads showed separation between reads from auricle fragments and reads from body groups as the first principal component, while the second principal component revealed variance between reads from clonal and non-clonal samples used in biological replicates of both the body and auricle groups (Supplementary Figure S1).

We identified human proteins with highest sequence conservation to *G. dorotocephala* sequences by performing BLASTX searches against the human reference proteome (GRCh38_latest_protein.faa) using the CLC Genomics Workbench. Gene Ontology analysis of identified human homologs was performed using PANTHER overrepresentation tests based on Fisher’s exact analysis (Mi et al., 2013) in the Gene Ontology Resource site (geneontology.org; Ashburner et al., 2000; The Gene Ontology Consortium, 2021).

We used Orthofinder version 2.5.1 (Emms and Kelly, 2019) to identify orthologs between our *G. dorotocephala* transcripts and the *S. mediterranea* transcripts from Ross et al. (2018). Orthofinder performs best when peptide sequences are used as input. We therefore used Transdecoder version 3.0.1 (https://github.com/TransDecoder) to translate the *S. mediterranea* and *G. dorotocephala* reference transcriptomes. We then identified *G. dorotocephala* transcripts that met the following criteria: (1) TPM >0.1, (2) *p*-value less than or equal 0.05, (3) fold-change of 5 or more, and (4) occurred in the same single-copy orthogroup as one of the 193 transcripts with reduced expression in *S. mediterranea* upon *SoxB1* RNAi (days 14 and 24 of RNAi) in Ross et al. (2018). To test whether the number of *G. dorotocephala* transcripts meeting these criteria was significant, we conducted a Monte Carlo analysis using a custom script available in the GitHub URL listed at the beginning of this section. Briefly, we randomly selected genes from the list of single-copy orthologs and counted how many of them occurred in the same single-copy orthogroup as one of the 193 transcripts with reduced expression in *S. mediterranea SoxB1* knockdowns. We ran this 10,000 times and counted the number of times we recovered overlaps greater than or equal to those found in our data.

### Imaging and Microscopy

Planarians and processed samples analyzed by bright and dark field microscopy, as well as those analyzed by low-magnification fluorescence microscopy, were photographed using an Axio Zoom V16 stereomicroscope (Zeiss, Oberkochen, Germany) equipped with an EOS Rebel T3 digital camera (Canon, Tokyo, Japan). High-magnification immunofluorescence analyses were carried out by confocal microscopy under a 10×, 20×, or oil-immersion 60× objective in a Nikon C2+ Confocal Microscope System. Z-stacks were generated from image sectioning of samples every 2– 3 microns and assembled using the NIS Elements Imaging Software (Nikon Corporation, Tokyo, Japan) to produce maximum projection and three-dimensional images. Brightness and contrast were adjusted for some images without producing changes that would alter interpretation of data.

## Results

### Auricle Morphology and Regeneration in *Girardia dorotocephala*

**FIGURE 1:**
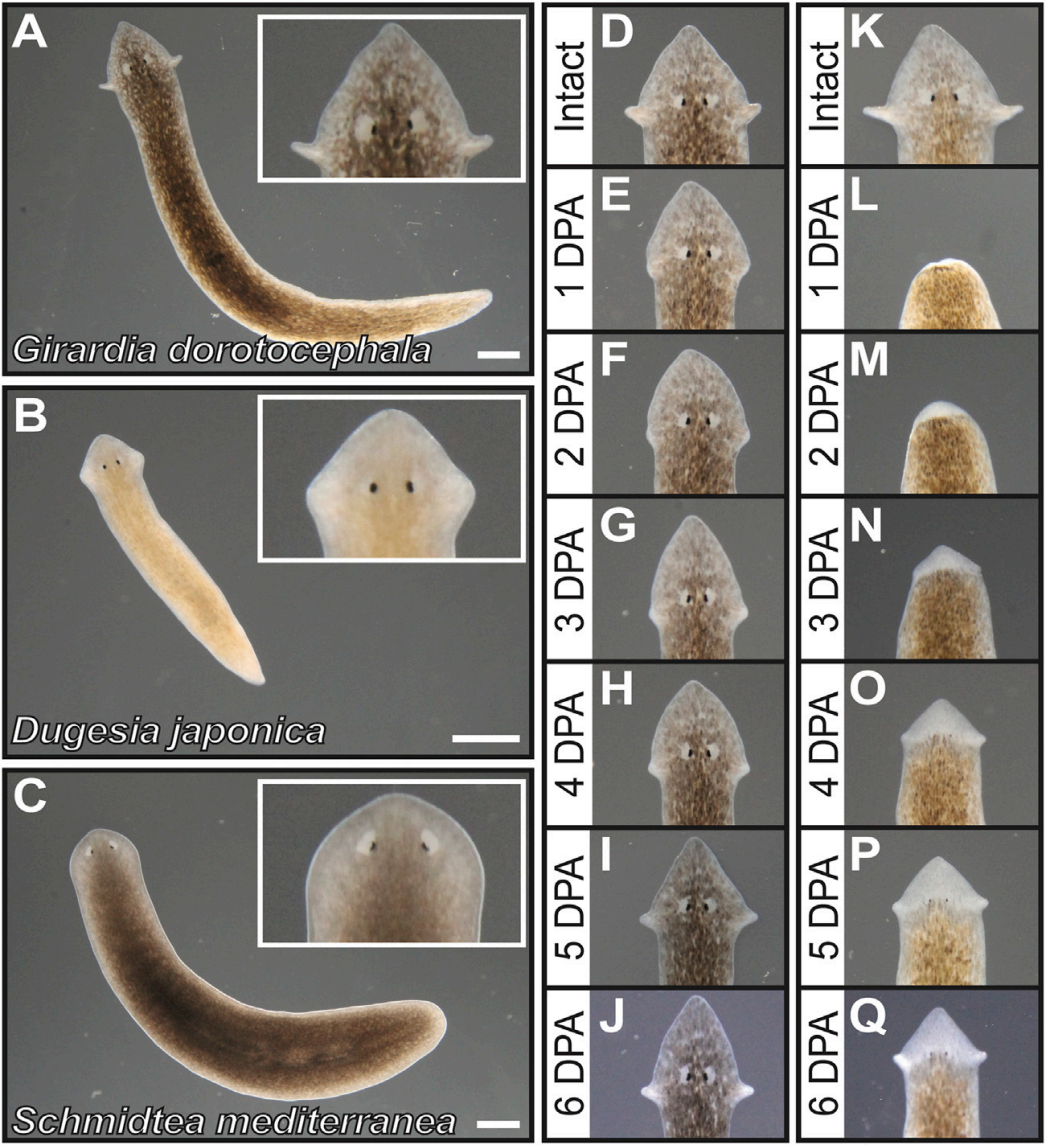
*Girardia dorotocephala* as research organisms for the study of auricle function and regeneration. **(A–C)** Dark = field images of live specimens of *G. dorotocephela* **(A)**, *Dugesia japonica* **(B)** and sexual biotype *Schmidtea mediterranea* **(C)**. **(D–Q)** Dark field images of *G. dorotocephela* undergoing auricle **(D–J)** and head **(K–Q)** regeneration. Images of intact **(D,K)** planarians, as well as daily timepoints from one to 6 days post-amputation (DPA) display the regenerative process that occurs within a week. Scale bars = 1 mm.

The North American planarian *Girardia dorotocephala* has distinctively pronounced auricle morphology in comparison to other planarian species that have been broadly adopted as laboratory organisms. Auricles in *G. dorotocephala* extend away from the rest of the head (Figure 1A), whereas auricles of *Dugesia japonica* are integrated within the proximal end of a triangular head structure (Figure 1B) and those of *Schmidtea mediterranea* are difficult to distinguish under low magnification microscopy (Figure 1C). Upon amputation of auricles from *G. dorotocephala*, tissue growth can be observed as early as 2 days post-amputation (2 DPA) and morphology that resembles the size and shape of original structures is distinguishable 5– 6 DPA (Figures 1D–J). Upon amputation of the entire head, initial formation of eye and auricle tissue can be observed under light microscopy 3–4 DPA and become clearly distinguishable 5–6 DPA (Figures 1J–Q).

**FIGURE 2:**
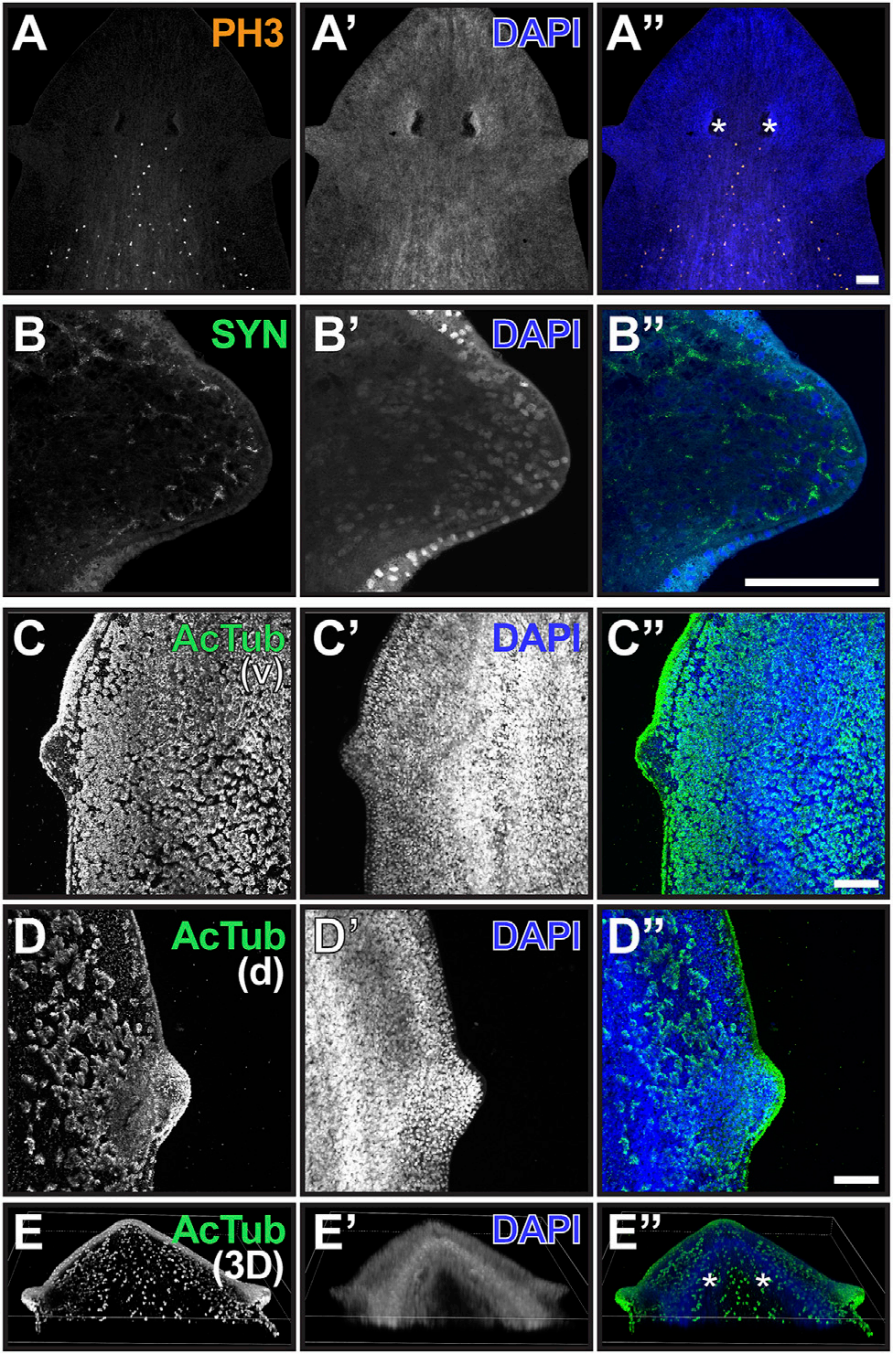
The auricle is largely composed of neurons and ciliated epithelia. **(A–D)** Maximum intensity projection of confocal z-stack images of whole-mount samples analyzed by phospho-Histone H3 Ser10 [PH3; **(A and A”)**], synapsin [SYN; **(B and B”)**], and acetylated alpha-tubulin [acTub; **(C and D)** and **(C” and D”)**] immunofluorescence. Ventral (v) and dorsal (d) views of ciliated epithelium are shown in **(C and D)**, respectively. DNA staining using DAPI reveals nuclei in **(A’–D’)**; blue in **(A”–D”)** and is used to visualize total cellular distribution. Scale bars = 0.1 mm. **(E)** Three-dimensional reconstruction of whole-mount sample analyzed as in **(D)** illustrates distribution of ciliated cells in the head of *G. dorotocephala*. Position of photoreceptors is marked by asterisks in **(A”,E”)**.

Although genetic markers to identify cells-types specific to the auricle are not available for *G. dorotocephala*, antibodies to highly conserved antigens from other species can be used to visualize some general features of these structures. Cells labeled by the mitotic M-phase marker Histone H3 phospho-Serine10 (PH3) are observed abundantly posterior to the eyes, but absent from the auricles and the anterior end of the planarian head (Figures 2A–A”). Given that neoblasts are the only actively dividing cells in the planarian soma (reviewed by Rink, 2013), this indicates that auricles are composed entirely of differentiated cells and non-mitotic neoblast progeny. Previous analysis of distribution of cells labeled by the conserved neoblast markers *GdPiwi1* and *GdPiwi2* corroborate with the interpretation that stem cells are absent from auricles and much of the head of *G. dorotocephala* (Almazan et al., 2018).

Visualization of structures recognized by the pan-neural marker anti-Synapsin (SYN; Klagges et al., 1996; Cebrià, 2008) revealed the presence of neuronal extensions throughout the interior of the auricle and reaching out to the most distal cell layer (Figures 2B,B”). Acetylated alpha-Tubulin antibodies (AcTub) labeled outer cell layer of the auricles (Figures 2C–E), where multiciliated epithelial cells with presumably motile cilia covered much of the lateral and dorsal anatomy of the auricle (Figures 2D,E). However, structures recognized by AcTub were largely absent from the ventral epithelium of the auricle (Figure 2C). This is surprising given that this antibody labels motile cilia of cells throughout much of the rest of the ventral epithelium of the planarian anatomy, which are known to propel gliding (Figures 2C,C”; Sanchez Alvarado and Newmark, 1999; Rompolas et al., 2010). A band of ciliated cells present along a dorsal midline that resembles structures recently shown to contribute to sensing of water flow (rheosensation) and vibrations in *S. mediterranea* (Ross et al., 2018) was also detected by AcTub immunofluorescence in *G. dorotocephala* (Figures 2E,E”), although dorsal ciliated cells dispersed between the auricle and the midline are also observed (Figures 2D,E).

### Auricle Amputation Results in Decreased Positive Chemotactic Ability that is Restored within 2 Days

**FIGURE 3:**
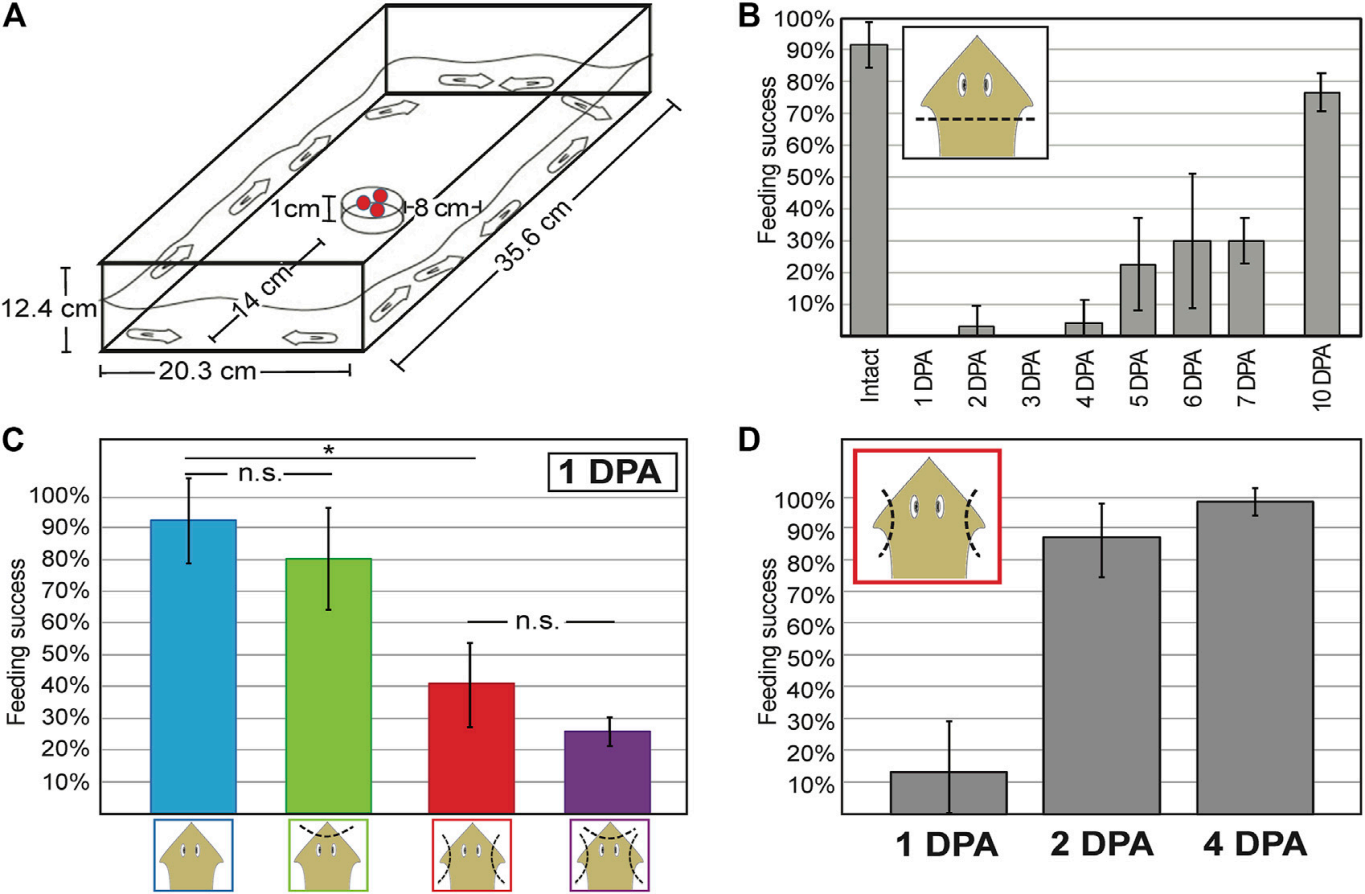
Decreased chemotactic response is observed after auricle amputation and restored 2 days post-amputation. **(A)** Dimensions of feeding arena used to measure chemotactic response. Pellets of liver mixed with agarose and food dye sit as chemoattractant on a petri dish in the center of the field. **(B)** Bar graph illustrating the average percent of intact and regenerating planarians on different days post-amputation (DPA) of the head (*x-*axis) that performed successfully in independent chemotaxis response assay trials (n ≥ 3 biological replicates) by the end of 30 min sessions. **(C)** Average percent feeding success of intact planarians (blue) and planarians subjected to either head tip (green) or auricle (red) amputation, or both (purple), at the end of 30-min chemotactic response assays performed 1-DPA. **(D)** Average percent feeding success of planarians 1, 2, and 4 days following auricle amputation (DPA) reveal recovery of chemotactic response within 48 h. Error bars illustrate standard deviation from the mean. Asterisks (*) represent Student’s t-test *p*-value ≤ 0.05. No statistical significance (n.s.) is indicated.

To examine the role of auricles in positive chemotaxis, behavioral response to liver (as chemical stimulant) was compared between intact planarians and planarians subjected to different types of amputations. Chemotaxis assays were performed in large (35.6 × 20.3 cm) feeding arenas with a Petri dish positioned as a pedestal to hold the liver 1 cm from the bottom of the arena (Figure 3A). The decision to position the liver at 1 cm height was based on the observation that elevating the stimulant as little as 5 cm decreases the ability of planarians to find food within a 1-h period (Supplementary Figure S2) in ways that are not observed when horizontal travel of similar distances is required (Figure 3). Under these conditions, intact *G. dorotocephala* were able to feed 90% of the time within a 30-min period (Figures 3B,C). In contrast, planarians subjected to complete head amputation failed to display significant feeding success during the first 4 days post-amputation (Figure 3B). Partial feeding success ranging from 20 to 30% in average was observed in planarians 5–7 DPA (Figure 3B). By 10 DPA feeding success recovered to above 75% (Figure 3B), which was not statistically significantly different from intact planarians (unpaired Student’s *t-*test > 0.05).

Planarians subjected to auricle amputation a day prior to assessment showed a significant decrease in chemotactic response (unpaired Student’s *t-*test < 0.05) and displayed 40% feeding success, whereas 90% of intact planarians tested displayed feeding success in parallel assays (Figure 3C). The decrease in behavioral response observed after auricle amputation in *G. dorotocephala* appears to be largely specific to positive chemotaxis, as no significant difference in traveling speed or time to acceleration after transfer were observed in separate tests 1-day after auricle amputation (Supplementary Figure S3). Analyses of negative chemotaxis using turmeric (Miyamoto et al., 2020; Supplementary Figure S3) and Allyl isothiocyanate (Arenas et al., 2017; data not shown) were inconclusive. To distinguish between changes in positive chemotactic response caused by the absence of auricles rather than general head injury, planarians were subjected to head tip amputation and head tip amputation in addition to auricle amputation. Both head tip amputee groups showed a 10% decrease in average chemotactic response when compared to their counterparts (intact vs. head tip amputation; auricle amputation vs. head tip and auricle amputation; Figure 3C). Because these differences were not statistically significant (unpaired Student’s *t-*test > 0.05) and only modest in comparison to auricle amputation, these findings suggest that auricles are specifically required for normal chemotactic ability towards positive stimulants.

To assess whether and when chemotactic response is restored after auricle amputation, groups of planarians were amputated 1, 2, and 4 days prior to testing for assessment on the same day. As seen in the initial analysis (Figure 3C), the majority of auricle-less planarians failed to show normal chemotactic response 1 DPA (Figure 3D; 40% average feeding success, unpaired Student’s *t-*test < 0.05). However, planarians tested 2 DPA and 4 DPA achieved approximately 90% feeding success within the allocated 30-min period (Figure 3D). The feeding success observed in 2 and 4 DPA amputees was comparable to that of intact planarians in previous analyses (Figures 3B,C), therefore indicating restoration of chemotactic ability. Altogether, these results show that loss of auricles leads to a significant reduction in positive chemotactic behavior which is restored within 48-h post-amputation.

### Neoblasts are Required for Anatomic Regeneration of the Auricle

**FIGURE 4:**
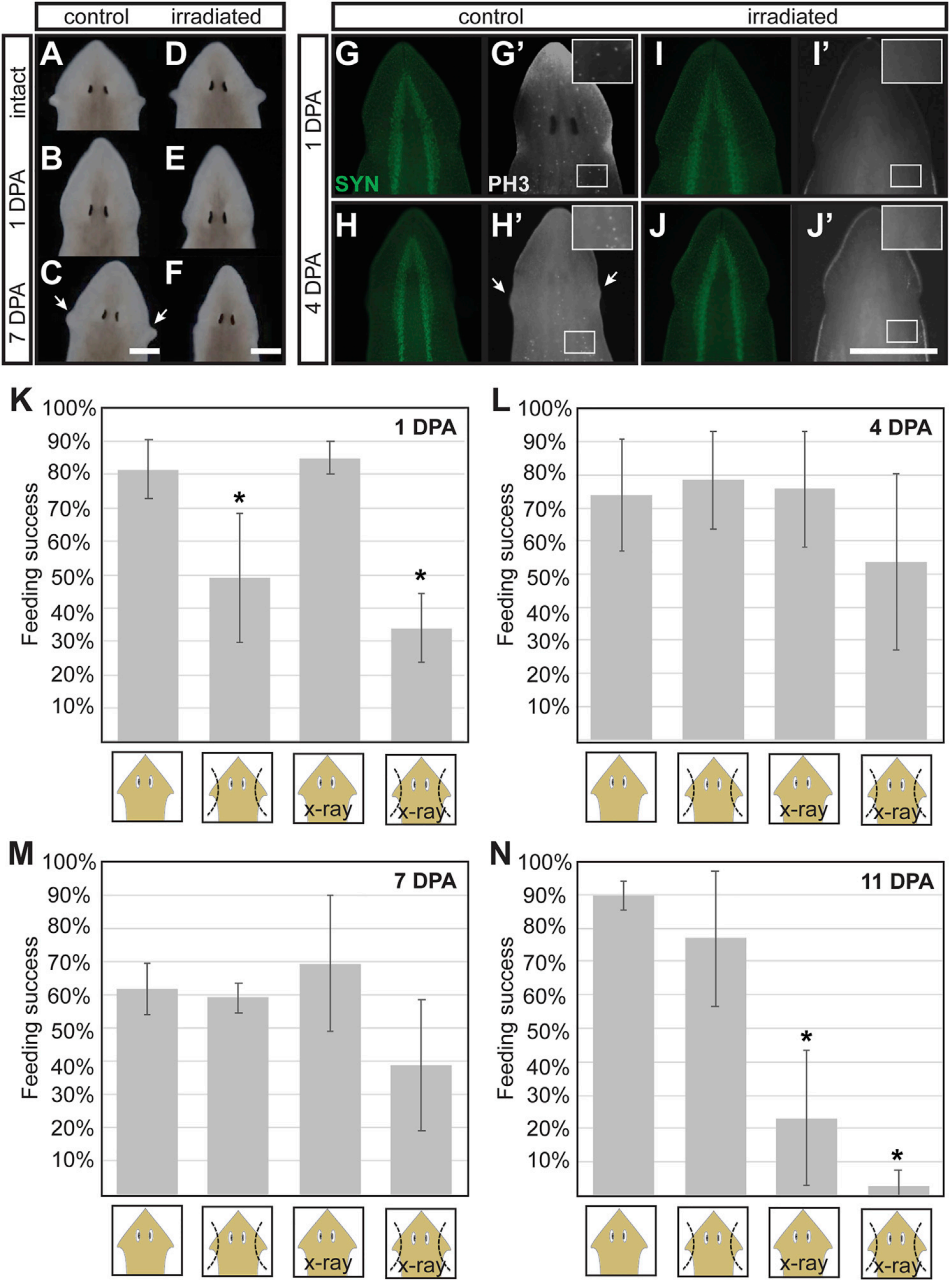
Stem cell requirements for morphological and full functional recovery after auricle amputation. **(A–F)** Darkfield microscopy images of control [not irradiated; **(A–C)**] and x-ray irradiated **(D–F)** *G. dorotocephala* shown before amputation **(A,D)**, 1 day post-amputation [DPA; **(B,E)**, and 7 DPA **(C,F)**] reveal that irradiated animals fail to regenerate the auricles regenerated by control samples 7DPA (arrows). **(G–J)** Images of planarians analyzed by immunofluorescence using anti-synapsin [SYT; green; **(G–J)**] and phospho-Histone H3 [PH3, **(G’–J’)**, white] antibodies reveal that mitotic stem cells present in control animals **(G’,H’)** are absent in irradiated samples **(I, J’)** while the overall structure of the central nervous systems (CNS) remains comparable **(G–J)**. **(K–L)** Average percent feeding success of intact (1st and 3rd bar in graphs) and auricle amputee (2nd and 4th bars per graph) control (1st and 2nd bars) and irradiated (X-ray; 3rd and 4th bars) planarian tested 1 **(K)**, 4 (L), 7 (M), and 11 (N) days post-amputation (DPA). Averages calculated from at least 3 biological replicate groups of at least 7 planarians each. Error bars indicate standard deviation from the mean. Asterisks (*) indicate statistical significance according to Students’ *t-*test *p-*value < 0.05 when compared to intact control samples.

To determine whether neoblasts contribute to the regeneration of auricle anatomy and the prompt restoration of chemotactic ability observed 2 days after auricle amputation, we measured the effect of x-ray irradiation on these processes. X-ray irradiation is routinely used as a chemical-free treatment to specifically deplete stem cells from planarian flatworms (Wolff and Dubois, 1948; Baguña et al., 1989; Shibata et al., 1999; Hayashi et al., 2006; Rouhana et al., 2010; Tasaki et al., 2016). It has been shown that mitotic neoblasts are selectively lost 1 day post-irradiation (1 DPI) and differentiating neoblast progeny within 2- and 3-DPI (Eisenhoffer et al., 2008). For our experiments, auricles were amputated from groups of *G. dorotocephala* subjected to x-ray irradiation 3 days prior, alongside a control group of non-irradiated planarians. The overall appearance of control and irradiated planarians was indistinguishable before auricle amputation (Figures 4A,D), as well as 1 day after auricle amputation (Figures 4B,E). However, control planarians were visibly able to regenerate their auricles 7 DPA (Figure 4C), whereas irradiated planarians failed to do so (Figure 4F). We assessed the integrity of the nervous system in irradiated planarians by immunostaining with SYN antibodies and verified that its overall morphology was undistinguishable between control and irradiated planarians (Figures 4G–J). Immunofluorescence using PH3 antibodies revealed that neoblasts were present in control planarians (Figures 4G’,H’) and absent in irradiated groups (Figures 4I’,J’), which validated the effectiveness of x-ray irradiation treatments. These results show that amputated auricles fail to regenerate in irradiated planarians, supporting the notion that auricle regeneration requires differentiation of stem cells and cannot be achieved by morphallaxis alone.

To determine whether reestablishment of normal chemotactic response after auricle amputation is driven by stem cells, control and irradiated planarians were subjected to feeding assays 1, 4, 7, and 11-days following auricle amputation. As observed in previous experiments, auricle amputation resulted in a significant decrease in feeding success 1 DPA, and this was observed in both irradiated and non-irradiated amputees (Figure 4K). Intact control and irradiated animals showed comparable feeding success, indicating that irradiation alone does not influence positive chemotactic ability under the used test conditions (Figure 4K). Unirradiated auricle amputees performed as well as intact control and irradiated planarians 4 DPA (Figure 4L). Irradiated amputees displayed feeding success which, although lower in average, was not significantly different to intact controls according to unpaired two-tailed Student’s *t-*tests (*p*-value > 0.05; Figure 4L). A similar trend was observed in planarians tested 7 DPA (Figure 4M). By 11 DPA irradiated intact and auricle-amputated planarians stopped eating (Figure 4N), most likely due to homeostatic decay caused by irradiation. These results show that x-ray irradiation has no direct effect on chemotactic behavior, and suggest that some chemotactic capacity can be restored after auricle amputation in the absence of stem cell-driven regeneration of complete auricle morphology.

### Detailed Analysis of *G. dorotocephala* Auricle Regeneration During the First 2 DPA

**FIGURE 5:**
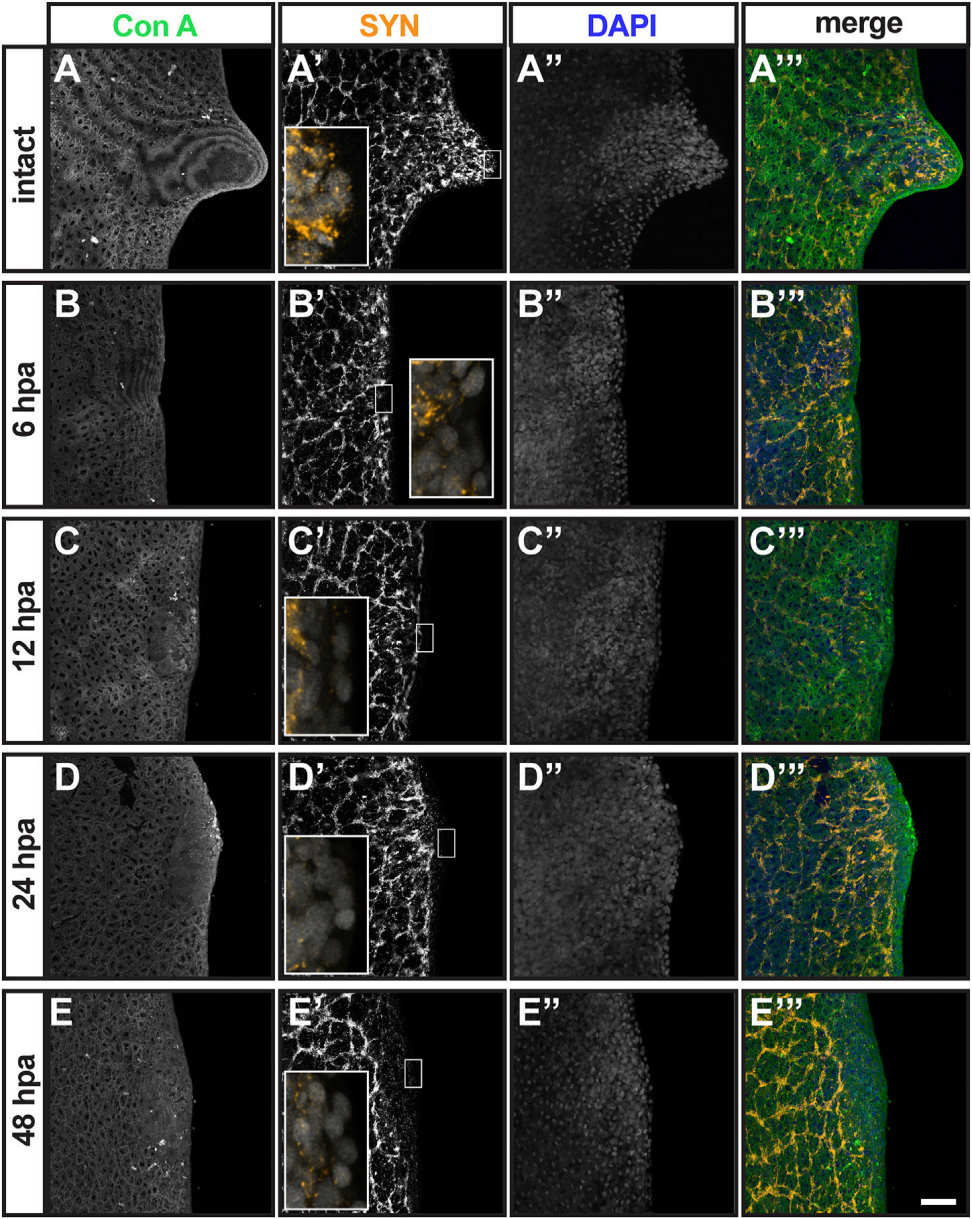
Analysis of epithelial and neuronal distribution during the first 2 days post-auricle amputation. Maximum intensity projection of confocal z-stack images from intact **(A–A”’)** and auricle amputees 6 h post-amputation [6 hpa; **(B–B”’)**, 12 hpa **(C–C”’)**, 24 hpa **(D–D”’)** and 48 hpa **(E–E”’)**], illustrate the distribution of epithelia stained with Concanavanil A [ConA; **(A–E)**; green in **(A”’–E”’)**] and neuronal projections stained with anti-synapsin [SYN; **(A’–E’)**; orange in **(A’–E’)** insets and **(A’”–E”’)**. DAPI staining of cell nuclei **(A”–E”)**; blue in **(A”’–E”’)**] reveals the general position of cells. Scale = 0.1 mm. Insets in **(A’–E’)** show 5-fold magnified views.

Thus far, auricles remain one of the least characterized structures in planarian flatworms. Generating new molecular markers for the study of planarian auricles is required for better understanding their development, function, and regeneration. With the tools available at this time, we attempted analyze the events that take place within the 2-day window when chemotactic ability is restored following auricle amputation (Figure 3D). First, we visualized wound healing by staining epithelial junctions in intact planarians and auricle amputees using Concanavilin A (ConA; Figures 5A–E). ConA was retained by epithelium present throughout the outer cell layer of the auricle anatomy in intact animals (Figure 5A). ConA-labeled epithelium also covered the area positioned for auricle regeneration in the earliest checked timepoint (6 h post-amputation; 6 HPA; Figure 5B) and throughout the analysis (12-, 24-, and 48-HPA; Figures 5C–E). This indicated that wound healing takes place during the first 6 hours following amputation and therefore is unlikely to be the last event required for restoration of chemotactic ability. Analyses using SYN antibodies revealed that neuronal extensions reached the outermost cellular layer of the intact auricle (Figures 2B, 5A). Upon amputation, the neuronal extensions labeled by SYN antibodies in the pre-existing tissue seemed to persist, and the developing auricle had decreased but detectable SYN signal (Figures 5B’–E’). No obvious differences were observed in SYN signal distribution at the position of auricle amputation between 24- and 48-HPA (Figures 5D’,E’), thus failing to reveal pivotal events in neurogenesis that could be responsible for restoration of chemotactic ability.

**FIGURE 6:**
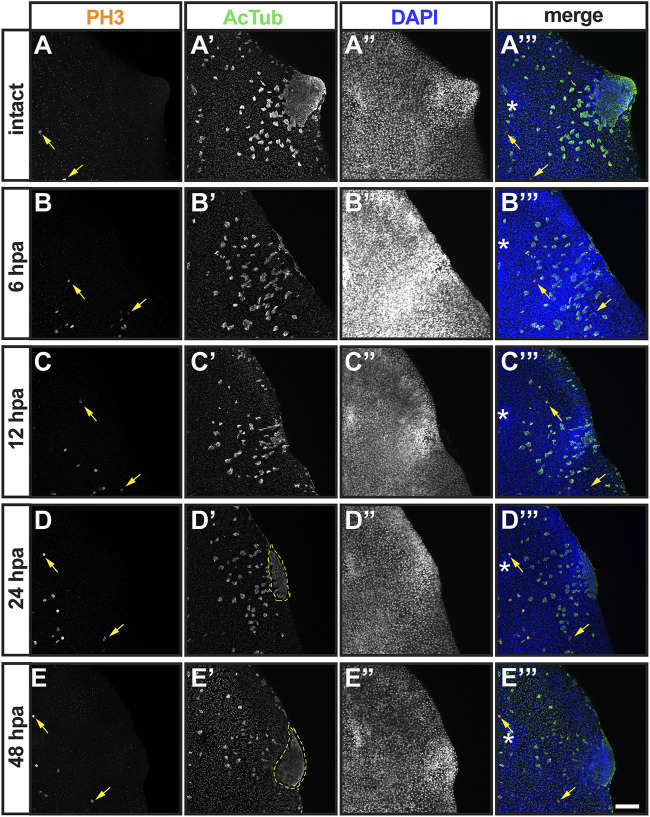
Analysis of mitotic and ciliated cell distribution during the first 2 days post-auricle amputation. Maximum intensity projection of confocal z-stack images from intact **(A–A”’)** and auricle amputees 6 h post-amputation 6 hpa; **(B, –B”’)**, 12 hpa **(C–C”’)**, 24 hpa **(D–D”’)** and 48 hpa **(E–E”’)**, illustrate distribution of M-phase neoblasts stained with phospho-Histone H3 [PH3; **(A–E)**; yellow arrows; orange in **(A”’–E”’)** and ciliated cells stained with anti-acetylated tubulin AcTub; **(A’–E’)**; green in **(A’–E’)** insets and **(A’”–E”’)**. DAPI staining of cell nuclei **(A”–E”)**; blue in **(A”’–E”’)** reveals the general position of cells. Area of aggregated ciliated cells (dashed lines) was quantified (Supplementary Material). Asterisks [* in **(A’”–E”’)**] mark position of eye. Scale = 0.1 mm.

To get a better idea of the timing when neoblast begin to give rise to replacement tissue, the distribution of M-phase cells close to the plane of amputation were visualized using PH3 antibodies. Previous studies have shown enrichment of PH3+ cells at the plane of amputation within 24 h of decapitation in *D. japonica* and *S. mediterranea*, which reach highest abundance in the 50 micron-region closest to the cut site 48 h post-amputation (Wenemoser and Reddien, 2010; Tasaki et al., 2011). More recent studies have shown that regeneration from smaller injuries, such as eye dissection, is achieved from existing progenitor cells without localized bursts in neoblast proliferation (LoCascio et al., 2017; Bohr et al., 2021). M-phase cells were rarely detected anterior to the photoreceptors in intact *G. dorotocephala* (average 2.8 PH3+ cells/sample; Supplementary Figure S4) and never within the auricle (Figure 2A; Figure 6A). Upon auricle amputation, accumulation of PH3+ cells on the plane of injury was not observed at any timepoint during the first 48 h following amputation (Figures 6B–E). The average number of mitotic cells anterior to the location of photoreceptors doubled at 12 HPA (2.8 vs 6.6 cells/sample; unpaired Student’s *t-*test < 0.05; Supplementary Figure S4), which may be indicative of a global burst in neoblast proliferation. These findings show that localized proliferation and accumulation of M-phase cells at the plane of injury does not take place during regeneration or amputated auricles, which suggests that post-mitotic neoblast progenitors migrate to the site of amputation to give rise to developing structures (as observed during eye excision in the work mentioned above).

Finally, we analyzed the presence and distribution of multiciliated epithelial cells at the position of auricle amputation using AcTub antibodies (Figure 6, A’–E’). As observed in our original analysis of intact planarians (Figures 2C–E), multiciliated cells fill the dorsal epithelia of the auricle and scatter in regions closer to the dorsal midline (Figure 6A’). Planarians analyzed 6 HPA displayed different patterns of cellular distribution (data not shown), from a few concentrated multiciliated epithelial cells at the site of amputation (n = 5/8) to a predominantly scattered cells (n = 3/8; Figure 6B’). We presume that this variability in distribution of multiciliated epithelial cells is partly due to inconsistencies in the precise location of amputation which are technically difficult to avoid. Similar variability was observed at 12 HPA (n = 4/10 scattered vs 6/10 concentrated; Figure 6C’) and at 1 DPA timepoints (n = 4/10 scattered vs 6/10 concentrated; Figure 6D’). At 2 DPA, all but one of the auricles analyzed a had concentrated multiciliated epithelial cells at the position of the regenerating structure (n = 9/10; Figure 6E’). In addition to the increase in number of samples with aggregated multiciliated cells at the position of auricle development, an increase in the area covered by these cells was noted. This observation was quantified by measuring the area of aggregated ciliated cells from maximum projections of z-stack images, which revealed that the region of concentrated multiciliated epithelial cells more than doubled between 1- and 2-DPA (Supplementary Figure S4). Although this difference did not reach statistical significance (Student’s *t-*test = 0.1), the correlation between trends suggests that accumulation of ciliated epithelia at the position of the auricle is a contributing factor in restoration of chemotactic ability between 1- and 2-DPA.

### Transcriptional Profiling of *G. dorotocephala* Auricles

The lack of expression markers currently available to study the cellular composition of *G. dorotocephala* auricles hinders our ability to analyze how differentiation of specific cell types (*e.g.* specific types of neurons, chemosensory cells) contributes to restoration of chemotactic behavior. In order to identify potential auricle-specific markers for future studies, as well as better characterize the cellular composition of planarian auricles, we performed transcriptomic analyses of these structures by Illumina RNA sequencing (RNAseq).

**FIGURE 7:**
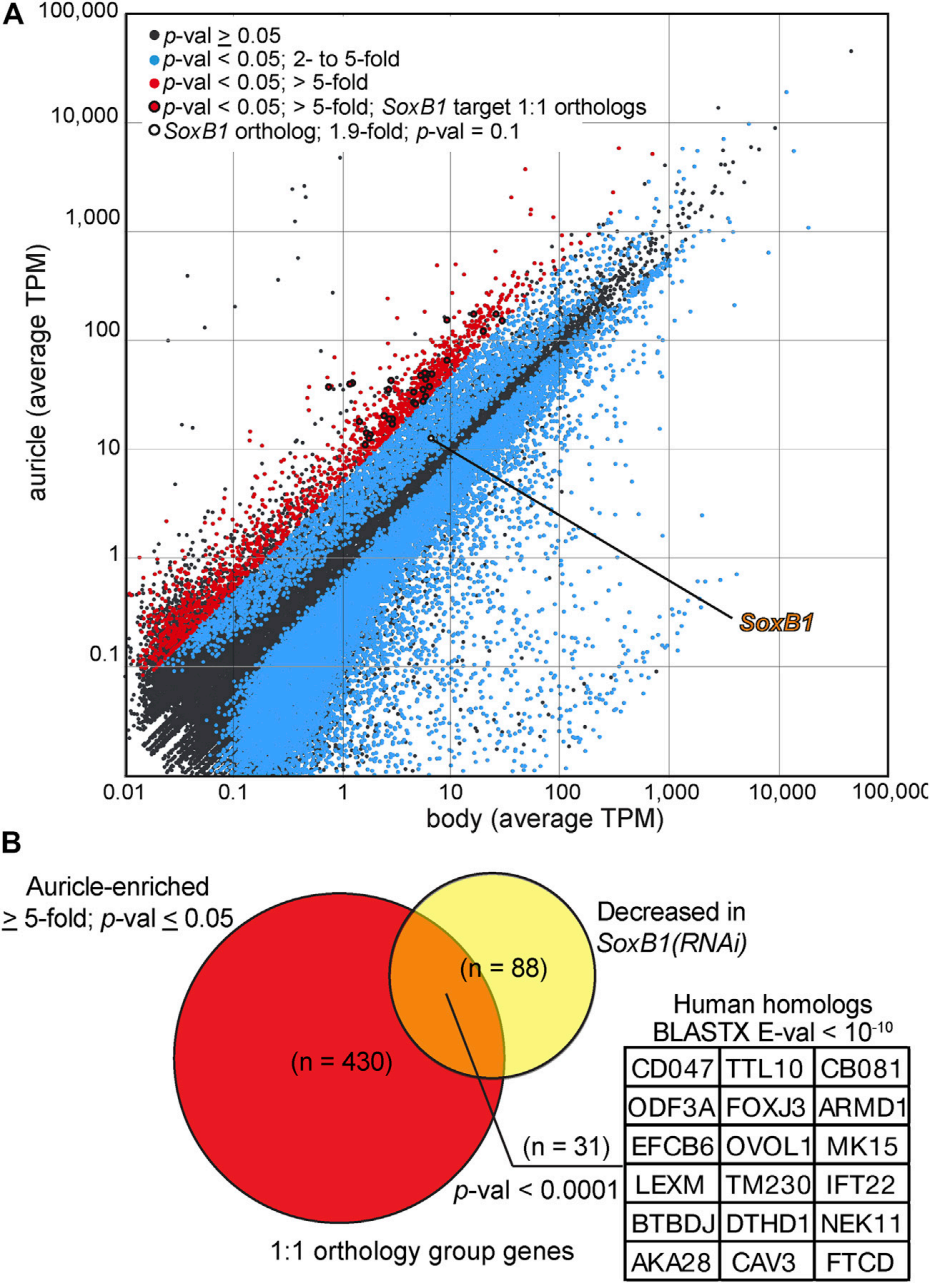
Analysis of auricle gene expression by RNAseq. **(A)** Log plot of average transcripts per million (TPM) calculated from Illumina reads of RNA extracted from *G. dorotocephala* auricles (*y* axis) and bodies (*x* axis) mapped to reference transcriptome contigs. Contigs with (Student’s *t-*test, *p-*val < 0.05; blue) and without (gray) statistically significant differences in relative gene expression are marked. Contigs with statistically significant difference in gene expression and ≥ 5-fold enriched abundance in reads of auricles are shown in red. Points representing the *G. dorotocephala* ortholog of *Smed-SoxB1* (white with black circumference) as well genes corresponding to those listed in panel **(B)** (red with black circumference) are highlighted. **(B)** Venn diagram showing the overlap of 1:1 orthologs between sequences enriched in the auricle (> 5-fold) of *G. dorotocephala* (red) and sequences with decreased expression 14- and 24-days into *Sox-B1* RNAi in *S. mediterranea* (Ross et al., 2018; yellow). Inset lists top human matches with E-value ≤ E10^-10^ from BLASTX searchers of *G. dorotocephala* sequences in orthology groups represented in the intersection of the Venn diagram.

We identified 39,737 contigs with significant differences in abundance (≥ 2-fold difference, *p-*value ≤ 0.05, minimal 0.1 cumulative TPM) between mapped reads from biological replicates of auricles and body fragments (Figure 7A; Supplementary Material S6 complete dataset available as Supplementary Material S2 at https://github.com/josephryan/Almazan_et_al_auricles_regen; see Materials and Methods section for details). A high threshold of ≥ 5-fold enrichment was applied to the group of differently-expressed genes to identify candidates with particularly favored auricle expression. This action revealed 1870 sequences (less than 0.7% of all reference contigs; Figure 7A, red). The percentage of sequences within this group of 1870 contigs that had strong conservation with human protein sequences (BLASTX E-value < 10^-2^) was enriched when compared to the entire reference transcriptome (33.8 vs. 12.1%; Supplementary Figure S5). GeneOntology (GO) analysis based on identified human homologs (BLASTX E-value < 10; n = 1440 uniquely mapped IDs) revealed enrichment of factors involved in cilia-related GO categories, such as outer and inner dynein arm assembly, sperm axoneme assembly, epithelial cilium movement, and regulation of cilium movement, as the most enriched biological processes (Supplementary Table S1). Genes involved in determination of left/right symmetry (False Discovery Rate (FDR) = 5.24E^−04^), regulation of cell projection organization (FDR = 2.86E^−02^, as well as neurogenesis (FDR = 2.31E^−02^) were also enriched GO biological processes. Surprisingly, “detection of chemical stimulus involved in sensory perception of smell” was the only GO biological process category that was significantly underrepresented amongst homologs of genes with ≥ 5-fold enriched expression in auricles (n = 12; 0.39-fold enrichment; FDR = 4.28E^−02^), suggesting that olfactory receptors genes are either highly divergent between these two species, less numerous in planarians, or without enriched expression in auricles of *G. dorotocephala*.

The identification of ciliary processes as top GO categories represented by genes with auricle-enriched expression (Supplementary Table S1) corroborated with the remarkable abundance of ciliated cells visualized by immunofluorescence in auricles of *G. dorotocephala* (Figure 2). A member of the Sox family of transcription factors (*Smed-SoxB1*) was recently shown to be required for the presence of ciliated sensory neurons in the auricles of the planarian *S. mediterranea* (Ross et al., 2018). The *G. dorotocephala* ortholog of *Smed-SoxB1* (TRINITY_DN4962_c1_g2) was enriched 1.9-fold in reads of auricles, but this enrichment did not reach statistical significance (*p-*value = 0.11; Figure 7A). Nevertheless, we identified significant overlap between the network of genes regulated by *Smed-SoxB1* and the collection of genes with enriched expression in auricles of *G. dorotocephala* identified by our RNAseq analysis. This was determined by first using Orthofinder (Martín-Durán et al., 2017; Emms and Kelly, 2019) to identify orthologs between our *G. dorotocephala* reference transcripts and the latest *S. mediterranea* reference transcriptome deposited in PlanMine (dd_Smed_v6; Rozanski et al., 2019). Our orthofinder analysis produced a set of 8,682 single copy orthologs. Of the 1,870 transcripts that had enriched abundance of 5-fold or more in *G. dorotocephala* auricles, 430 were in the list of single copy orthologs. Of these 430, 31 matched one of the 88 *Schmidtea* genes that were both in the 193 set of dd_Smed_v6 transcripts with decreased abundance upon *Smed-SoxB1-2* RNAi (14- and 24-day timepoints; Ross et al., 2018) as well as in the set of single copy orthologs (Figure 7B; Supplementary Table S2). Importantly, this set of 31 genes includes many factors not categorized as being involved in ciliary processes. To see if this was significant, we ran a Monte Carlo simulation where we randomly chose 430 genes from the list of 8,682 single copy orthologs and counted how many of them overlapped with the single copy orthologs from *SoxB1-*dependent genes. In zero out of the 10,000 iterations did we find 31 overlaps. In fact, the highest overlap in control iterations was 13 transcripts, which indicates that there is significant correlation (*p-*value ≤ 0.0001) between genes expressed with *SoxB1* dependence in *S. mediterranea* and genes expressed in cells that compose the auricles of *G. dorotocephala*.

Finally, we asked whether our list of highly-expressed auricle genes (enriched *≥* 5-fold) included components of developmental signaling pathways that could to provide insight into the mechanisms that drive formation of prominent auricles in *G. dorotocephala*. We examined auricle-enriched genes that fall under the GO category of “pattern specification process,” which is cataloged under the GO biological process group “determination of left/right asymmetry” (both of which were over-represented in genes with highly-enriched expression in auricles; Supplementary Table S1). Amongst fifty-seven genes under GO group “pattern specification process” (Supplementary Table S3), highly conserved homologs of Noggin (BLASTX = 1.29E^−12^), BMP-4 (BLASTX = 8.49E^−27^), and WNT2B (BLAST = 1.78E^−88^) had greater than 6-fold enriched expression in auricles of *G. dorotocephala.* Further assessment of these genes using functional approaches may determine the mechanisms underlying auricle development and the evolution of auricle morphology.

## Discussion

Here we show that amputation of *G. dorotocephala* auricles reduces foraging success in a laboratory setting, which corroborates with observations by Koehler (1932) and Asano et al. (1998) suggesting that auricles contain chemoreceptors that are crucial for normal feeding behavior (reviewed by Fraenkel and Gunn, 1961). Given that a liver/agarose mixture (rather than live prey) was used, and water flow was not a factor, the contribution of auricles to feeding success in our experiments can be attributed to chemical sensing. Surprisingly, the reduction in chemotactic ability observed after auricle removal was restored just 2 days after amputation, which is earlier than what was observed during similar experiments in *D. japonica* (Asano et al., 1998), and sooner than the time that it takes to regenerate the characteristically stretched anatomy of *G. dorotocephala* auricles (Figure 1D). Nevertheless, 2 days were enough for ciliated cells on the dorsal side of regenerating auricles to accumulate (Figure 6E), suggesting that bilateral detection of attractant concentrated at the position of the auricles may be crucial for orientating these animals during foraging. Interestingly, recovery was also observed in x-ray irradiated animals, which suggests that if accumulation of ciliated cells at the position of auricle amputation is indeed driving recovery, then these cells may come (at least in part) from pre-existing cells and/or post-mitotic progenitors, as is known to occur after dissection of planarian eyes (LoCascio et al., 2017).

Close interaction between two ciliated cell types, epithelial cells and presumed chemosensory cells of the subepidermis, was observed in analyses of *D. tigrina* auricles by electron microscopy (MacRae, 1967). These presumed chemosensory cells were described to possess 1-2 cilia that project between epithelial cells onto the outer surface, with the potential to directly reach chemoattractants (MacRae, 1967). MacRae noted that cilia from epithelial cells and those projecting from the subepidermis contain subtle differences in width and membrane composition (inferred from different reaction to fixatives). We were unable to distinguish between these separate populations of cilia with our methods. However, the recent study by Ross et al. (2018) revealed that *Smed-SoxB1* function is required for development of subepidermal multiciliated cells characterized as sensory neurons that populate much of the surface of the auricle in *S. mediterranea*. These cells express additional genes whose function are required for normal chemotactic behavior (*i.e. eml-1*, *pdka-1*, *Smed-37835*, and *sargasso-1*; Ross et al., 2018), corroborating with the idea that at least some of the cilia of auricles comes from sensory cells and not regular epithelia. Interestingly, *eml-1*, *pdka-1*, *Smed-37835*, and *sargasso-1* are not only expressed in cells at the position of auricles, but also along almost the entire circumference of the head and the rest of the animal, as well as in cells that mimic the distribution of ciliated cells in the dorsal midline. These observations suggest that the position of chemosensory cells expands well beyond the head.

**FIGURE 8:**
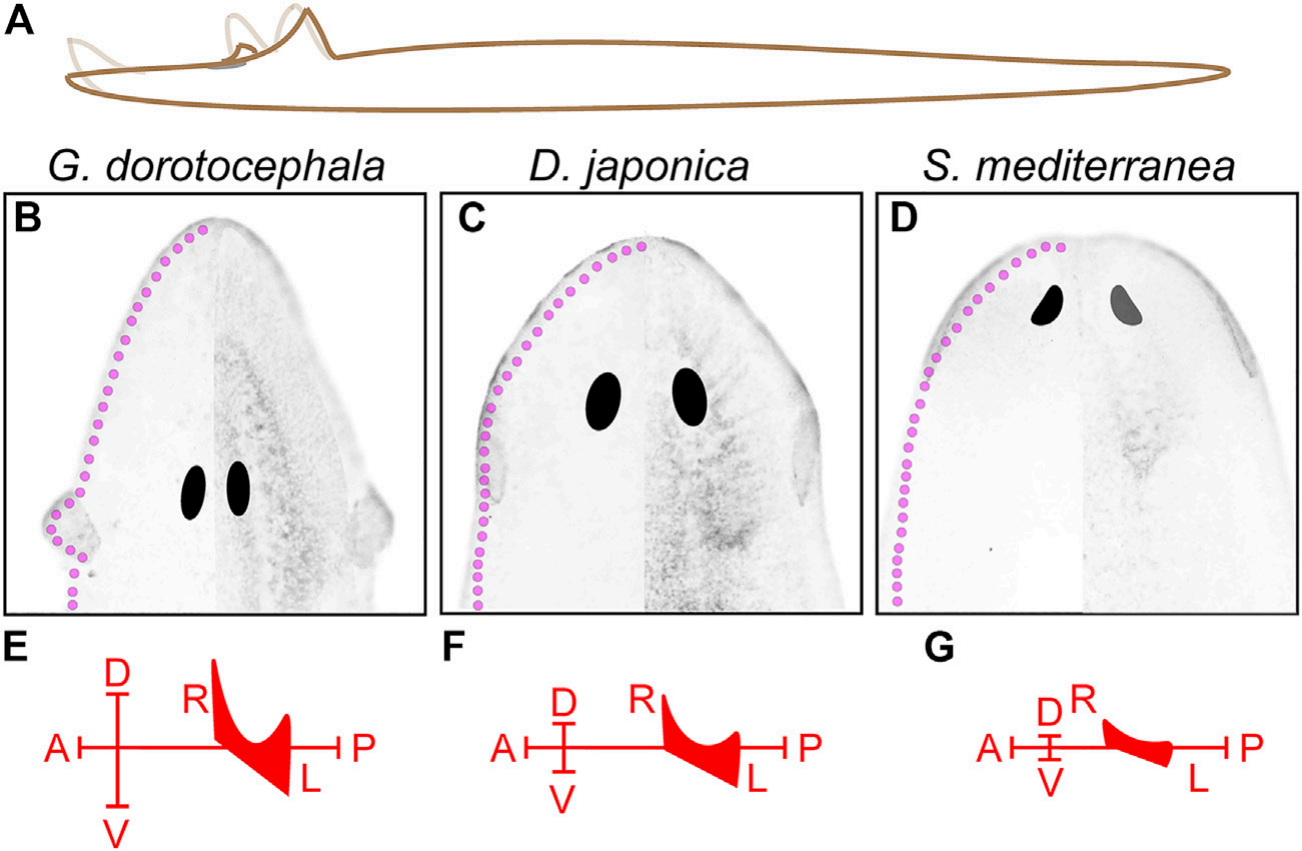
Possible contributions to chemical sensing from three-dimensional extension of auricle architecture. **(A)** Illustration of observed *G. dorotocephala* movements depicts the possible extension of 3-dimensional range covered by the auricles and head-tip. **(B–D)** Image of acetylated-alpha-tubulin-labeled ciliated sensory cells in *G. dorotocephala* **(B)**, *D. japonica* **(C)**, and *S. mediterranea* **(D)** are shown with the morphology and position of the central nervous system obtained from DAPI-stained samples is superimposed on the right half of each image. The hypothetical position of peripheral sensory cells (magenta) based on studies of *Gt-wnt5* in *G. tigrina* (Marsal et al., 2003), and TRP family genes in *D. japonica* (Inoue et al., 2014) as well as *S. mediterranea* (Arenas et al., 2017) are drawn along the entire periphery of the head of each species (magenta). **(E–G)** Hypothetical chemosensory axes established by the 3-dimensional distribution of sensory cells in each planarian species.

A separate study identified a *friend leukemia integration 1* homolog in *S. mediterranea* (*Smed-fli-1*) whose function is also required for foraging, but whose expression is distributed in much of the planarian brain branches as well as in a heterogeneous population of neurons close to the edge of the entire planarian head (Roberts-Galbraith et al., 2016). The observation that the genes required for positive chemotaxis that were identified by these two groups are expressed throughout most of the edge of the head in *S. mediterranea* suggests that chemotaxis is not entirely dependent on the auricles. Our own analyses show that, although concentrated in the auricles, multiciliated cells are present throughout the edge of the head in *G. dorotocephala* (Figure 2E). In addition, the partial feeding success observed 1-day post-amputation of auricles (Figures 3C,D), supports the notion that chemoreception during foraging also occurs elsewhere in the body. Perhaps the most extreme example to support this notion comes from recent experiments showing that the pharynx of the planarian *D. japonica* is able to find food on its own over short distances, and may even direct foraging behavior of the entire organism (Miyamoto et al., 2020). If indeed chemosensory cells are broadly distributed throughout the entirety of the animal circumference, as well as along the dorsal midline and the pharynx, then these may establish sensory gradients along anteroposterior and mediolateral axes, whereas auricles with prolonged architecture (as the ones observed in *G. dorotocephala*) may provide an additional structural element that enhances the animal’s ability to sense gradients along the animal’s dorsoventral and mediolateral axes in complex three-dimensional ecosystems (Figure 8).

Given the inconclusive results regarding restoration of chemotactic behavior in irradiated planarians (Figure 4), we are unable to predict how much of the behavioral recovery is due to rearrangements of pre-existing tissue as compared to stem cell-driven development of sensory neurons. Although the hypotheses that are more strongly supported by the data mentioned above are that chemical sensing is distributed throughout the entire animal, and that pre-existing non-mitotic cells may contribute to restoration of chemotaxis after auricle amputation, we have not ruled out the possibility that there are unique chemoreceptors in the auricle. Two days would be enough time for missing cell-types to be restored, based on the observation that changes in gene expression in neoblasts and early-neoblast progeny occur just within hours of injury (Gurley et al., 2010; Wenemoser et al., 2012; Wurtzel et al., 2015), and the appearance of new photoreceptor cells as early as 2-days following eye dissection (Deochand et al., 2016; Scimone et al., 2020). It is possible that accumulation of ciliated cells at the position of the auricle reestablishes an important chemosensory axis 2-DPA. However, it is also possible that specific chemosensory cells need to develop in the regenerating auricle. After all, the food used in our assays, and that found in nature, contains a multitude of potential chemoattractants that may trigger different planarian sensory cells. Thus far, large-scale screens for general neuronal (Cebria et al., 2002a; Cebria et al., 2002b; Nakazawa et al., 2003; Roberts-Galbraith et al., 2016) and specific sensory cell markers (Ross et al., 2018), as well as extensive single-cell RNAseq analyses (Wurtzel et al., 2015; Molinaro and Pearson, 2016; Fincher et al., 2018; Plass et al., 2018) have failed to identify genes exclusively expressed auricles of *S. mediterranea*. It seems worthwhile to pursue single-cell RNAseq and *in situ* hybridization screens in *G. dorotocephala*, which has more prominent auricule structures. A recent report using *D. japonica* showed auricular expression of a gene that is not broadly present in the head periphery but is also expressed in the pharynx (*Dj_fibroblast growth factor (Djfgf)*; Auwal et al., 2020). It is predicted that *Djfgf* expression provides positional information during regeneration, but its actual function remains unknown (Auwal et al., 2020). Nevertheless, the regional expression of *Djfgf* within the head of *D. japonica* suggests the presence of auricle-specific cell types. Analysis of *Djfgf* ortholog(s) in *G. dorotocephala*, as well as highly enriched genes in our auricle RNAseq analysis, could help determine whether cell types exclusive to the auricle exist in planarians.

Koehler’s seminal work in the study of the auricle included observations comparing the behavior of planarians seeking food in lentic and lotic ecosystems, and predictions that chemosensory cells must be present throughout the planarian body (Koehler, 1932). Ninety years later, researchers in the field are revisiting these questions and finding that chemotaxis and rheosensation may be take place in the same group of cells, or at least in cells with shared molecular programs (*i.e.* gene expression regulated by *SoxB1*; Ross et al., 2018). Variability in auricle morphology may provide physical attributes that optimize flow and capture of chemicals in specific habitats. In other words, the vast array of auricle morphologies observed in different planarian species may be due to selective pressures unique to each of their ecosystems (e.g. water flow, depth of habitat, position and distance relative to food) or differences in innate behavior [e.g. head tilting, preference for travel on curved or vertical surfaces, or spontaneous wigwag movements, as observed by Akiyama et al. (2015; 2018)]. Our analysis of *G. dorotocephala*, supports the notion that auricles do contribute significantly to chemotactic behavior, although partial recovery of their structure is sufficient for functional restoration under our tested laboratory conditions. It is possible that full auricle development is required for optimal detection of chemoattractants in the more complex three-dimensional space present in their natural habitats.

## Data Availability

The datasets presented in this study can be found in online repositories. The names of the repository/repositories and accession number(s) can be found in the Materials and Methods/Supplementary Material.
