# Supplementary figures and images for: Regeneration of Planarian Auricles and Reestablishment of Chemotactic Ability
Source: Front Cell Dev Biol. 2021 Nov 26;9:777951. doi: 10.3389/fcell.2021.777951 (PMC8662385; doi:10.3389/fcell.2021.777951)

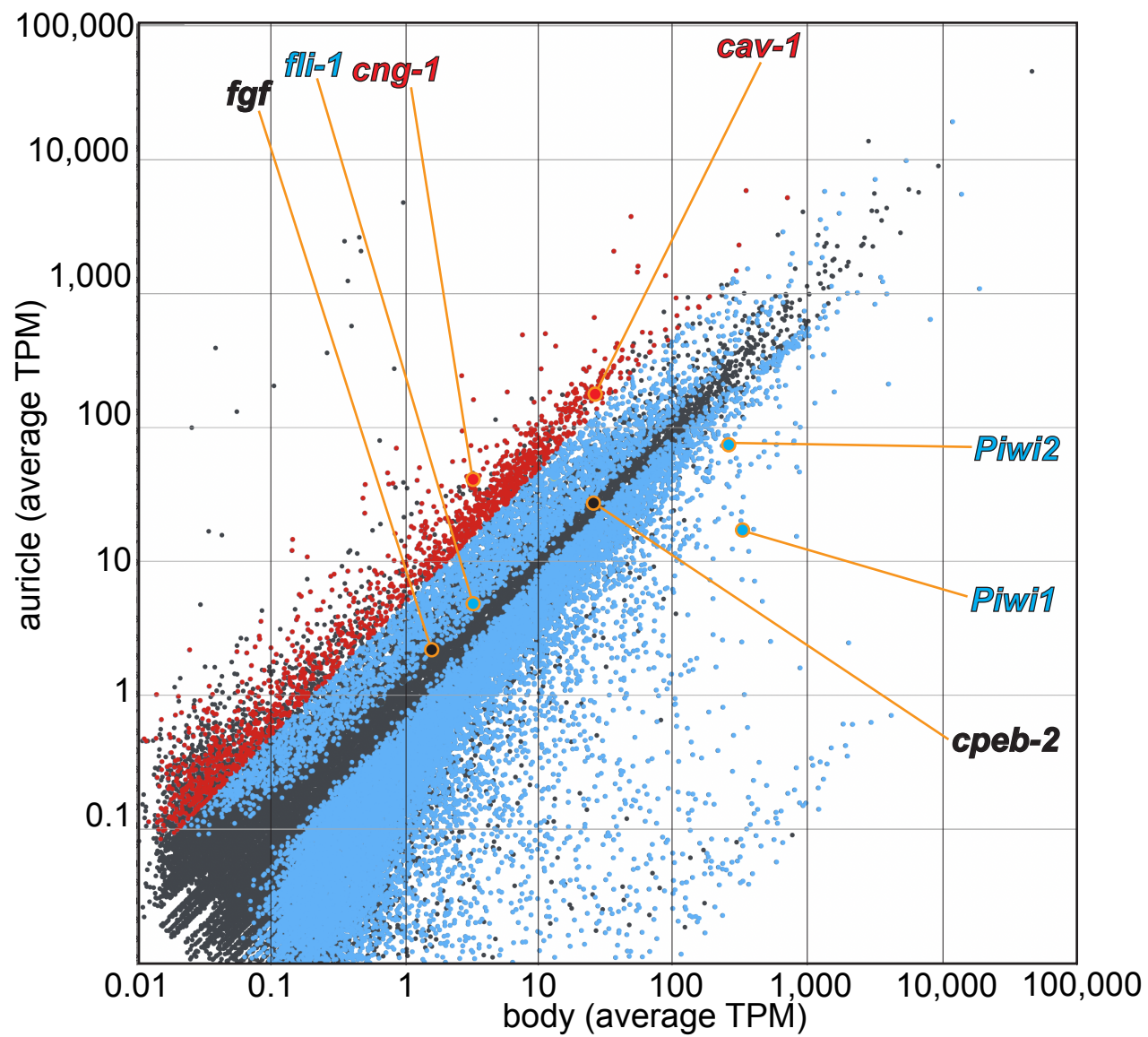

Supplement: Supplementary file 1 [file DataSheet7.PDF]

Principal component scatter plot

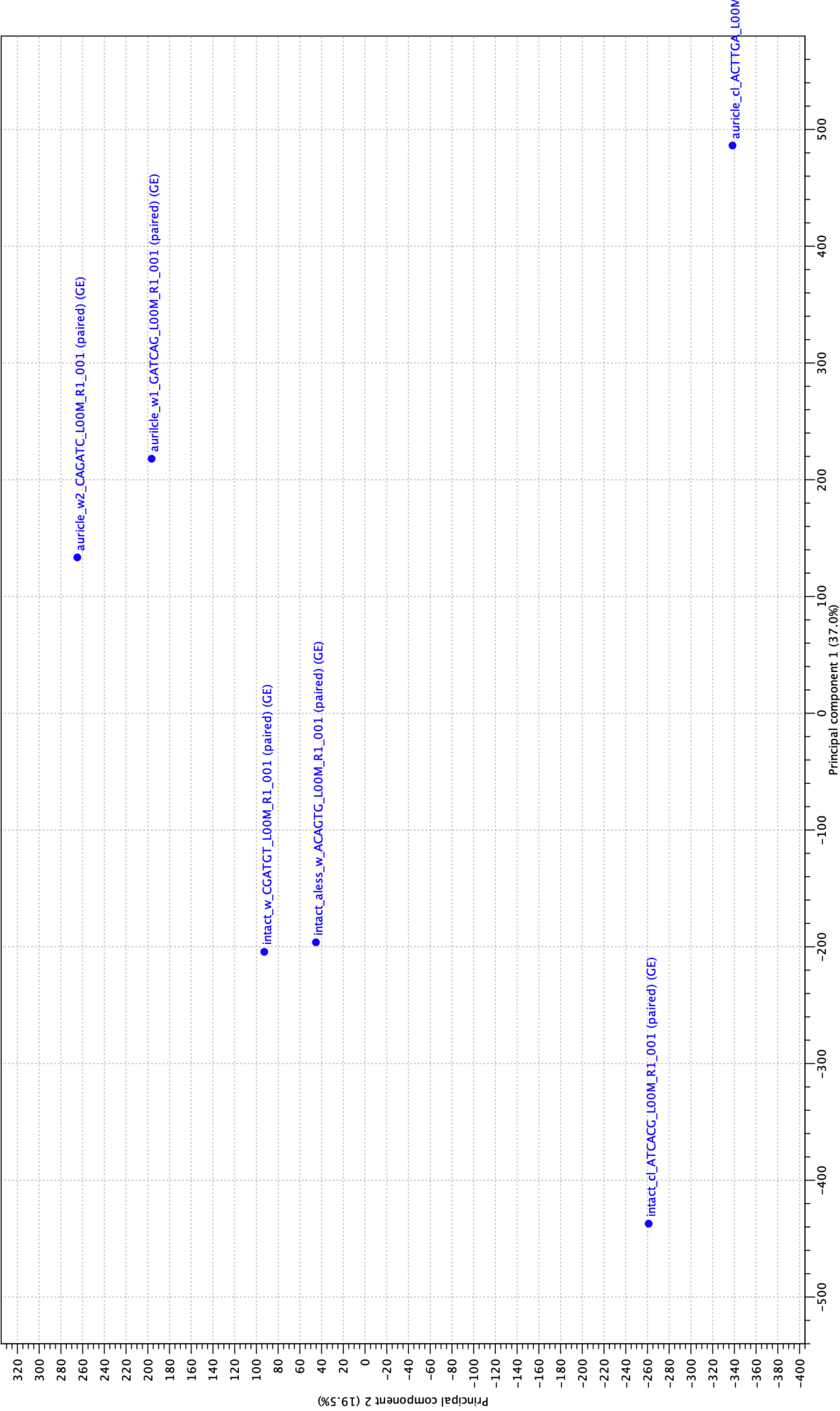

Supplement: Supplementary file 2 [file DataSheet2.PDF]

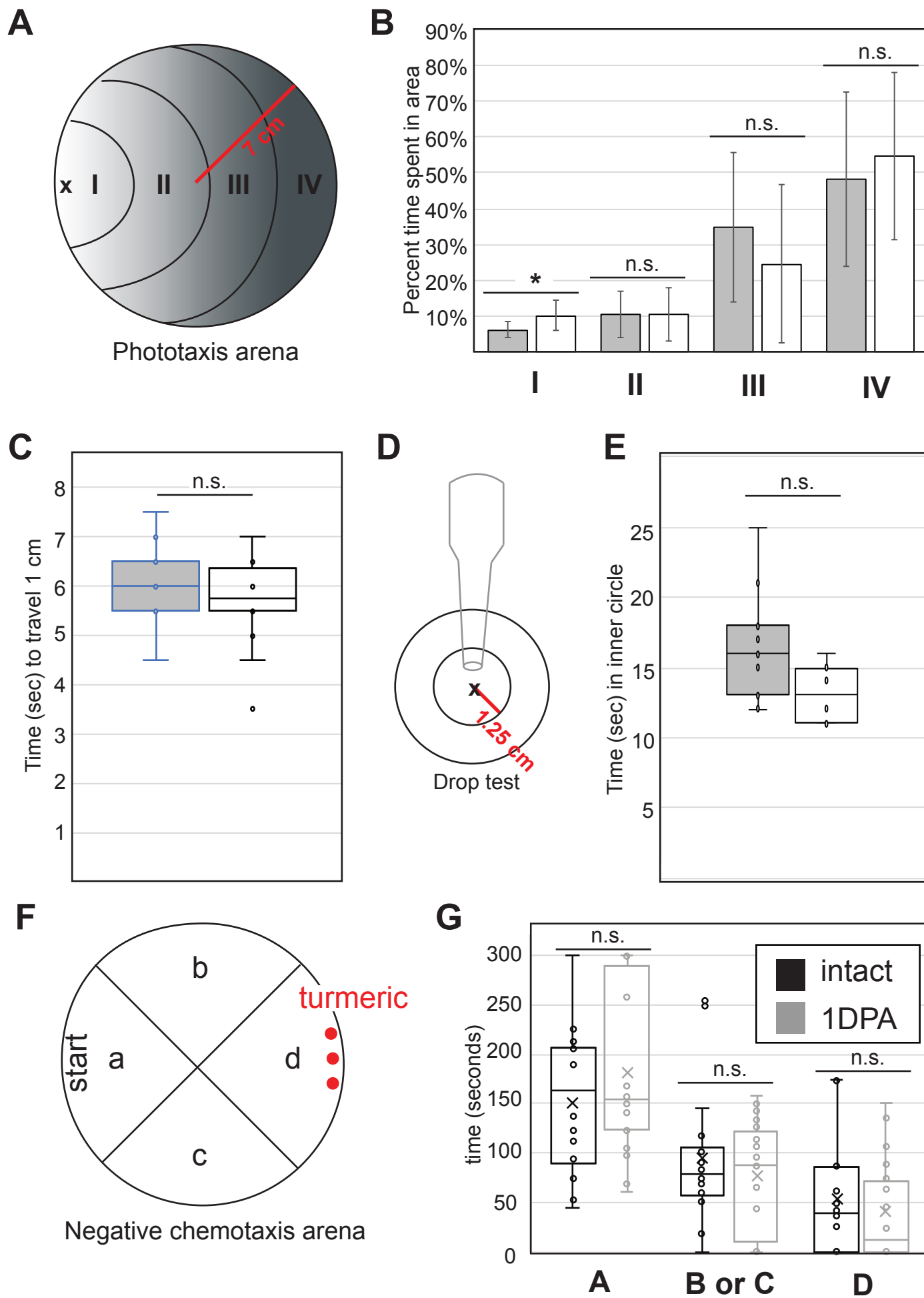

Supplement: Supplementary file 3 [file DataSheet4.PDF]

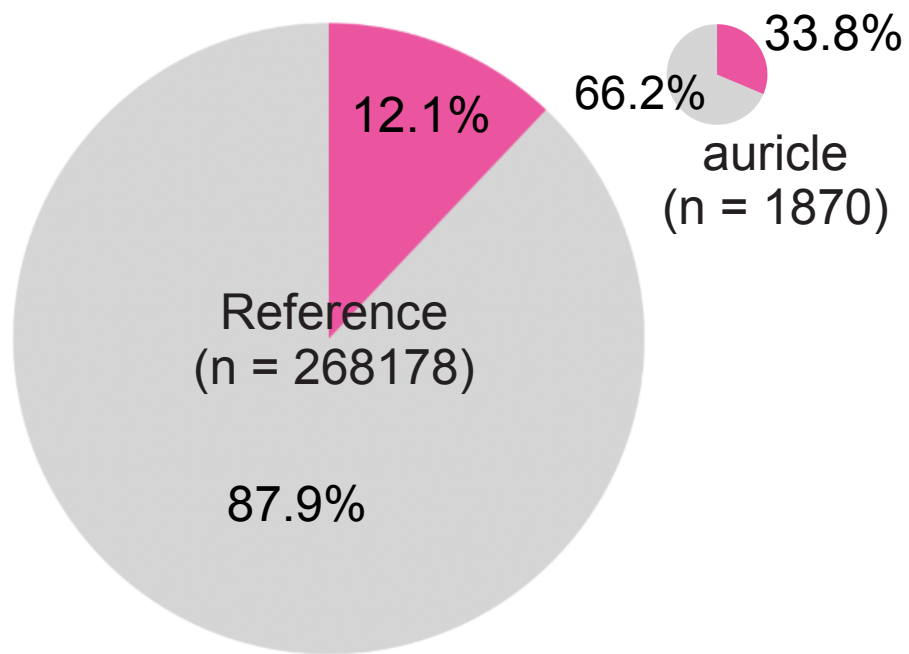

Human homologs (E-value < 10E<sup>-2</sup>)

Supplement: Supplementary file 4 [file DataSheet6.PDF]

**A**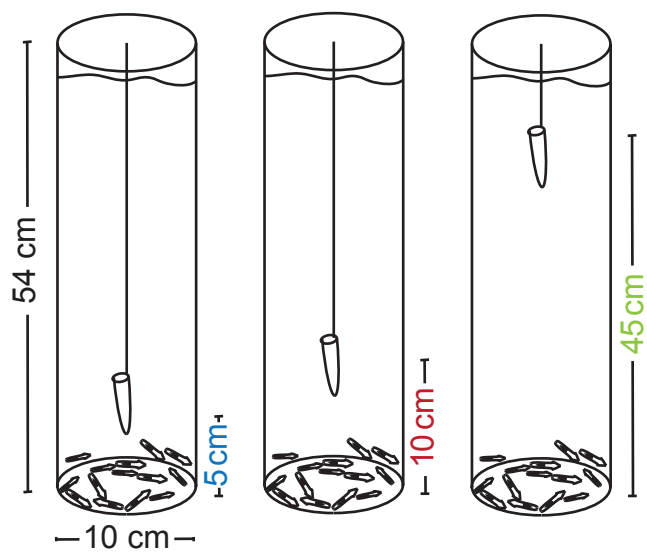**B**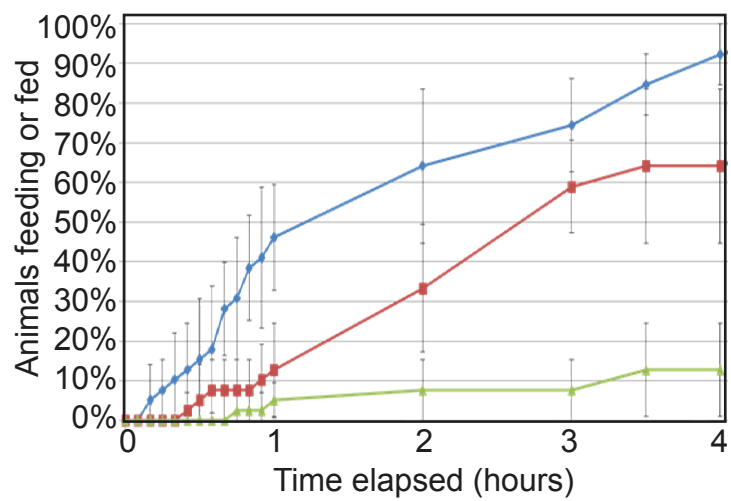

Supplement: Supplementary file 7 [file DataSheet3.PDF]

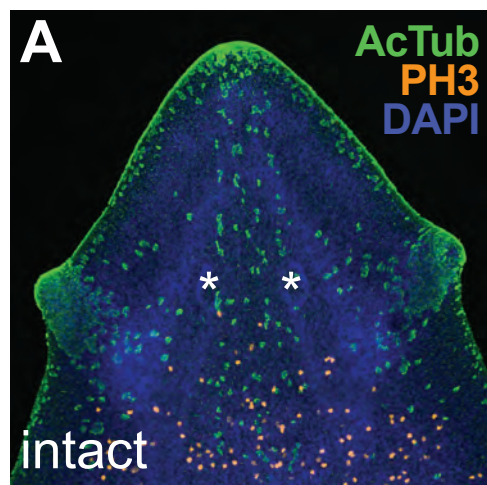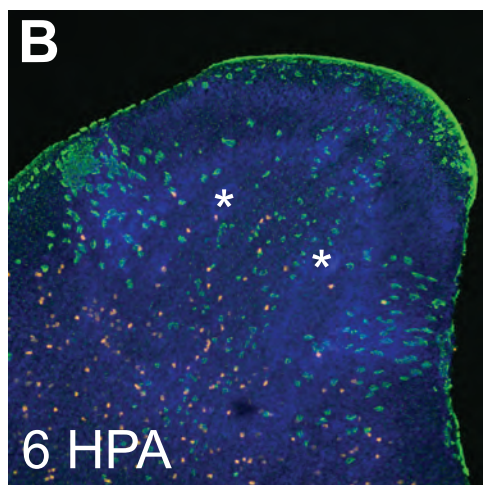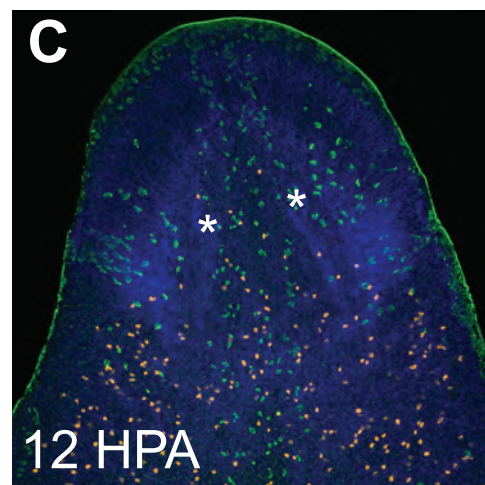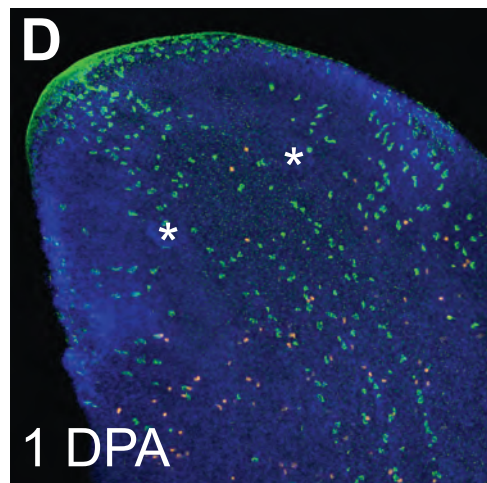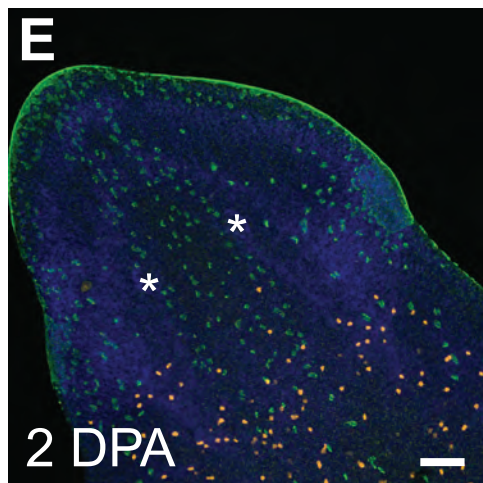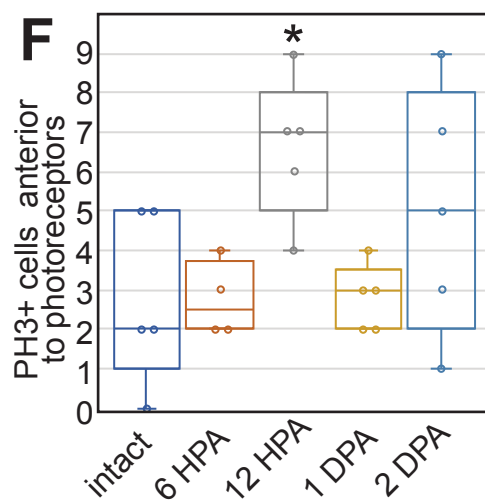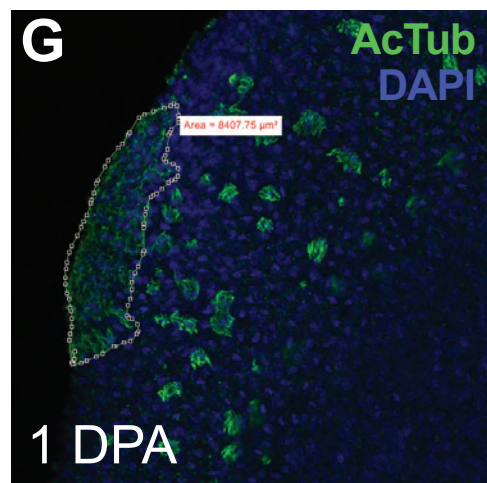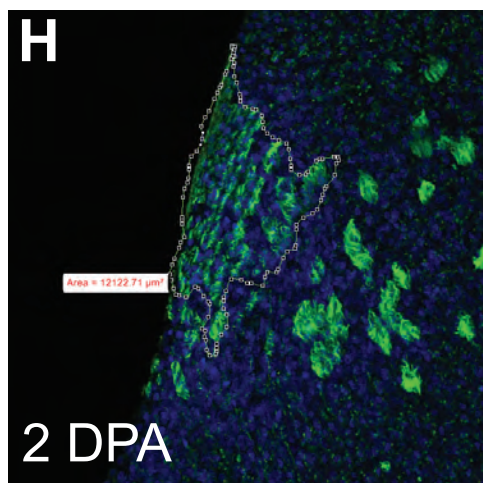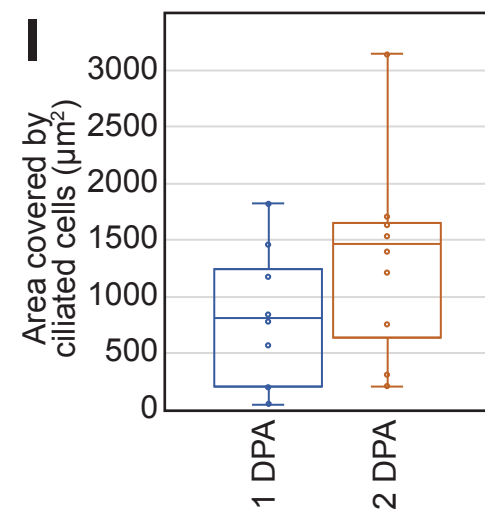

Supplement: Supplementary file 10 [file DataSheet5.PDF]
