# Supplementary material for: Regeneration of Planarian Auricles and Reestablishment of Chemotactic Ability
Source: Front Cell Dev Biol. 2021 Nov 26;9:777951. doi: 10.3389/fcell.2021.777951 (PMC8662385; doi:10.3389/fcell.2021.777951)
Supplement: Supplementary file 5 [file Table1.PDF]

Supplementary Table S1: Enriched GO groups amongst homologs of auricle-enriched transcripts.

GO biological process	Fold Enrichment	Raw P value	FDR
**outer dynein arm assembly**	7.80	8.24E-07	3.16E-04
**inner dynein arm assembly**	7.15	9.32E-05	1.93E-02
**sperm axoneme assembly**	6.87	2.32E-06	7.28E-04
**epithelial cilium movement involved in extracellular fluid movement**	6.13	4.02E-07	1.66E-04
**regulation of cilium movement**	4.40	8.46E-05	1.77E-02
**cerebral cortex cell migration**	3.90	2.20E-04	4.06E-02
**nucleoside diphosphate phosphorylation**	3.39	2.36E-04	4.26E-02
**determination of left/right symmetry**	3.11	1.60E-06	5.24E-04
**(pattern specification process)**	1.92	1.44E-05	3.60E-03
**cytoskeleton-dependent intracellular transport**	2.29	1.35E-04	2.59E-02
**heart development**	1.77	3.40E-05	7.75E-03
**protein phosphorylation**	1.76	3.32E-06	9.14E-04
**cell morphogenesis involved in differentiation**	1.69	2.22E-04	4.05E-02
**regulation of cell projection organization**	1.62	1.53E-04	2.86E-02
**neurogenesis**	1.42	1.14E-04	2.31E-02
