# Supplementary material for: Regeneration of Planarian Auricles and Reestablishment of Chemotactic Ability
Source: Front Cell Dev Biol. 2021 Nov 26;9:777951. doi: 10.3389/fcell.2021.777951 (PMC8662385; doi:10.3389/fcell.2021.777951)
Supplement: Supplementary file 6 [file Table2.PDF]

Supplementary Table S2. *G. dorotocephala* auricle-enriched orthologs of genes with decreased expression upon extended *SoxB1* RNAi in *S. mediterranea*

G. dorotocephala Supercontig	*S. mediterranea* ortholog	Human match - Description	Human match - Accession	BLASTX E-value	Fold change	*p*-value
**TRINITY_DN5787_c0_g1**	dd_Smed_v6_12022_0_1	CD047_HUMAN UPF0602 protein C4orf47 OS=Homo sapiens OX=9606 GN=C4orf47 PE=2 SV=1	A7E2U8	9.824E-30	50.8751	0.0165
**TRINITY_DN108137_c0_g1**	dd_Smed_v6_13656_0_1	RHG20_HUMAN Rho GTPase-activating protein 20 OS=Homo sapiens OX=9606 GN=ARHGAP20 PE=1 SV=2	Q9P2F6	0.0555507	33.3288	0.0019
**TRINITY_DN21130_c0_g1**	dd_Smed_v6_8903_0_1	ODF3A_HUMAN Outer dense fiber protein 3 OS=Homo sapiens OX=9606 GN=ODF3 PE=1 SV=1	Q96PU9	4.312E-42	17.1515	0.0119
**TRINITY_DN2245_c0_g1**	dd_Smed_v6_13336_0_1	PLXA1_HUMAN Plexin-A1 OS=Homo sapiens OX=9606 GN=PLXNA1 PE=1 SV=3	Q9UIW2	1.07649	15.2208	0.0240
**TRINITY_DN4392_c0_g1**	dd_Smed_v6_11972_0_1	CNBD2_HUMAN Cyclic nucleotide-binding domain-containing protein 2 OS=Homo sapiens OX=9606 GN=CNBD2 PE=2 SV=2	Q96M20	0.0531169	13.5746	0.0125
**TRINITY_DN9519_c0_g1**	dd_Smed_v6_14102_0_1	CLIP3_HUMAN CAP-Gly domain-containing linker protein 3 OS=Homo sapiens OX=9606 GN=CLIP3 PE=1 SV=3	Q96DZ5	2.59126	12.6478	0.0181
**TRINITY_DN689_c0_g1**	dd_Smed_v6_3220_0_1	AAKB1_HUMAN 5'-AMP-activated protein kinase subunit beta-1 OS=Homo sapiens OX=9606 GN=PRKAB1 PE=1 SV=4	Q9Y478	3.73802	10.9312	0.0186
**TRINITY_DN1688_c0_g1**	dd_Smed_v6_7631_0_1	EFCB6_HUMAN EF-hand calcium-binding domain-containing protein 6 OS=Homo sapiens OX=9606 GN=EFCAB6 PE=1 SV=1	Q5THR3	8.193E-21	9.1861	0.0202
**TRINITY_DN7318_c0_g1**	dd_Smed_v6_10776_0_1	LEXM_HUMAN Lymphocyte expansion molecule OS=Homo sapiens OX=9606 GN=LEXM PE=2 SV=4	Q3ZCV2	4.63E-29	8.7424	0.0071
**TRINITY_DN34421_c0_g1**	dd_Smed_v6_11553_0_1	BTBDJ_HUMAN BTB/POZ domain-containing protein 19 OS=Homo sapiens OX=9606 GN=BTBD19 PE=2 SV=1	C9JJ37	1.234E-22	8.5392	0.0016
**TRINITY_DN5872_c0_g1**	dd_Smed_v6_9117_0_1	AKA28_HUMAN A-kinase anchor protein 14 OS=Homo sapiens OX=9606 GN=AKAP14 PE=1 SV=1	Q86UN6	3.583E-14	8.4568	0.0086
**TRINITY_DN14000_c0_g1**	dd_Smed_v6_10509_0_1	CRCC2_HUMAN Ciliary rootlet coiled-coil protein 2 OS=Homo sapiens OX=9606 GN=CROCC2 PE=1 SV=4	H7BZ55	2.99275	8.2928	0.0183
**TRINITY_DN53286_c0_g1**	dd_Smed_v6_14858_0_1	TTL10_HUMAN Inactive polyglycylase TTLL10 OS=Homo sapiens OX=9606 GN=TTLL10 PE=1 SV=2	Q6ZVT0	3.28E-86	7.7876	0.0120
**TRINITY_DN9284_c0_g1**	dd_Smed_v6_9995_0_1	not available	not available		7.7668	0.0224
**TRINITY_DN2216_c0_g2**	dd_Smed_v6_9787_0_1	GUCNB_HUMAN Putative uncharacterized protein GUCA1ANB OS=Homo sapiens OX=9606 GN=GUCA1ANB PE=5 SV=2	X6R8D5	0.958996	7.4823	0.0158
**TRINITY_DN3601_c1_g1**	dd_Smed_v6_13009_0_1	FOXJ3_HUMAN Forkhead box protein J3 OS=Homo sapiens OX=9606 GN=FOXJ3 PE=1 SV=2	Q9UPW0	3.78E-17	7.4114	0.0235
**TRINITY_DN12218_c0_g1**	dd_Smed_v6_12010_0_1	OVOL1_HUMAN Putative transcription factor Ovo-like 1 OS=Homo sapiens OX=9606 GN=OVOL1 PE=1 SV=3	O14753	1.403E-51	7.2084	0.0005
**TRINITY_DN5000_c0_g1**	dd_Smed_v6_7770_0_1	UPP_HUMAN Uracil phosphoribosyltransferase homolog OS=Homo sapiens OX=9606 GN=UPRT PE=1 SV=1	Q96BW1	7.50266	7.2075	0.0194
**TRINITY_DN15076_c0_g2**	dd_Smed_v6_11371_0_1	TM230_HUMAN Transmembrane protein 230 OS=Homo sapiens OX=9606 GN=TMEM230 PE=1 SV=1	Q96A57	1.686E-11	7.0618	0.0167
**TRINITY_DN6834_c0_g1**	dd_Smed_v6_15379_0_1	DTHD1_HUMAN Death domain-containing protein 1 OS=Homo sapiens OX=9606 GN=DTHD1 PE=2 SV=3	Q6ZMT9	3.786E-15	6.8041	0.0208
**TRINITY_DN5283_c0_g1**	dd_Smed_v6_8555_0_1	CAV3_HUMAN Caveolin-3 OS=Homo sapiens OX=9606 GN=CAV3 PE=1 SV=1	P56539	2.244E-20	6.7974	0.0135
**TRINITY_DN14351_c0_g1**	dd_Smed_v6_9924_0_1	CB081_HUMAN Uncharacterized protein C2orf81 OS=Homo sapiens OX=9606 GN=C2orf81 PE=3 SV=3	A6NN90	4.57E-19	6.5712	0.0090
**TRINITY_DN14179_c0_g1**	dd_Smed_v6_18133_0_1	ARMD1_HUMAN Armadillo-like helical domain containing protein 1 OS=Homo sapiens OX=9606 GN=ARMH1 PE=2 SV=2	Q6PIY5	2.374E-120	6.5616	0.0056
**TRINITY_DN13547_c0_g1**	dd_Smed_v6_5692_0_1	ZBT40_HUMAN Zinc finger and BTB domain-containing protein 40 OS=Homo sapiens OX=9606 GN=ZBTB40 PE=1 SV=4	Q9NUA8	1.74327	6.1854	0.0036
**TRINITY_DN662_c0_g1**	dd_Smed_v6_8567_0_1	not available	not available		6.1012	0.0149
**TRINITY_DN1022_c0_g1**	dd_Smed_v6_7005_0_1	not available	not available		5.9051	0.0068
**TRINITY_DN1632_c0_g1**	dd_Smed_v6_6769_0_1	MK15_HUMAN Mitogen-activated protein kinase 15 OS=Homo sapiens OX=9606 GN=MAPK15 PE=1 SV=1	Q8TD08	5.461E-138	5.8758	0.0119
**TRINITY_DN2527_c0_g1**	dd_Smed_v6_11176_0_1	IFT22_HUMAN Intraflagellar transport protein 22 homolog OS=Homo sapiens OX=9606 GN=IFT22 PE=1 SV=1	Q9H7X7	6.494E-48	5.5355	0.0081
**TRINITY_DN3602_c0_g1**	dd_Smed_v6_9098_0_1	NEK11_HUMAN Serine/threonine-protein kinase Nek11 OS=Homo sapiens OX=9606 GN=NEK11 PE=1 SV=2	Q8NG66	9.655E-92	5.3023	0.0024
**TRINITY_DN8746_c0_g1**	dd_Smed_v6_7815_0_1	FTCD_HUMAN Formimidoyltransferase-cyclodeaminase OS=Homo sapiens OX=9606 GN=FTCD PE=1 SV=2	O95954	2.949E-109	5.2274	0.0160
**TRINITY_DN7860_c0_g1**	dd_Smed_v6_19428_0_1	not available	not available		5.0083	0.0189
