# Supplementary material for: Regeneration of Planarian Auricles and Reestablishment of Chemotactic Ability
Source: Front Cell Dev Biol. 2021 Nov 26;9:777951. doi: 10.3389/fcell.2021.777951 (PMC8662385; doi:10.3389/fcell.2021.777951)
Supplement: Supplementary file 8 [file Table3.PDF]

Supplementary Table S3. Homologs of genes with enriched expression in auricles belonging to GO: “pattern specification process”

Fold Change	*p*-value	Gene name; Gene symbol	BLASTX E-value
2.9929E+11	0.023580445	Semaphorin-3A;SEMA3A	3.25281
2.06738E+11	0.019733989	Transcription factor HES-4;HES4	8.11941
2.04943E+11	0.004430949	Bone morphogenetic protein 7;BMP7	1.5558
1.23313E+11	0.009407586	Neurogenic differentiation factor 1;NEUROD1	2.89567
1.05421E+11	0.009407586	Homeobox protein Hox-D8;HOXD8	0.576027
22.73795842	0.001413612	Transcription factor Sp8;SP8	1.3E-50
19.9526738	0.009742205	Reelin;RELN	2.94325
18.32431472	0.000452778	Fez family zinc finger protein 2;FEZF2	1.14E-83
15.95913826	0.021472137	Bone morphogenetic protein 4;BMP4	8.49E-27
12.79900061	0.043371629	Adenomatous polyposis coli protein 2;APC2	0.400259
12.62436779	0.041709744	Transcriptional repressor protein YY1;YY1	0.353388
10.58077033	0.007871958	Noggin;NOG	1.29E-12
10.07961739	0.028151502	Bone morphogenetic protein receptor type-2;BMPR2	3.33964
9.423250038	0.004861785	Transcription factor LBX1;LBX1	7.26E-21
8.990093908	0.02226978	Zinc finger protein ZIC 3;ZIC3	1.86E-45
8.155406202	0.007434841	Enkurin;ENKUR	3.64E-70
8.132790133	0.021290483	UPF0691 protein C9orf116;C9orf116	1.84E-07
8.066416671	0.014583153	Coiled-coil domain-containing protein 151;CCDC151	2.12E-69
8.044813059	0.00811574	Integrin alpha-M;ITGAM	1.66974
7.962144119	0.01811088	Tetratricopeptide repeat protein 25;TTC25	2.97E-111
7.790941042	0.012625013	Cilia- and flagella-associated protein 52;CFAP52	0
7.758105391	0.011039419	Protein pitchfork;PIFO	6.65E-17
7.628464255	0.023992113	Cilia- and flagella-associated protein 53;CFAP53	2.88E-22
7.555684477	0.027544877	Pro-neuregulin-3, membrane-bound isoform;NRG3	9.04E-03
7.532061278	0.048640457	Proprotein convertase subtilisin/kexin type 5;PCSK5	0.930242
7.515281676	0.011966079	Homeobox protein DLX-1;DLX1	1.03E-14
7.382757674	0.029935888	Meiosis-specific nuclear structural protein 1;MNS1	6.86E-64
7.365459721	0.009246414	Dynein intermediate chain 2, axonemal;DNAI2	0
7.251810003	0.016958682	Cytoplasmic dynein 2 heavy chain 1;DYNC2H1	4.98853
7.17874146	0.011750056	Tetratricopeptide repeat protein 8;TTC8	0.995836
7.016391192	0.000283495	Complement C3;C3	0.107743
6.811229372	0.01109765	Dynein intermediate chain 1, axonemal;DNAI1	0
6.805531943	0.049796372	E3 ubiquitin-protein ligase MIB1;MIB1	6.31E-34
6.485791318	0.021722365	Intraflagellar transport protein 25 homolog;HSPB11	1.08E-29
6.47386426	0.021847987	Armadillo repeat-containing protein 4;ARMC4	0
6.440599567	0.012642696	Protein Wnt-2b;WNT2B	1.78E-88
6.425170712	0.037346227	Dynein regulatory complex protein 1;DRC1	1.4E-137
6.263474729	0.010669604	Homeobox protein aristaless-like 4;ALX4	2.1E-30
6.179680848	0.035864027	Polycystic kidney disease protein 1-like 1;PKD1L1	7.83122
6.179680846	0.035864027	Chondroitin sulfate synthase 1;CHSY1	9.88825
6.171373911	3.88013E-06	COUP transcription factor 2;NR2F2	2.25E-95
6.16603494	0.008438441	Low-density lipoprotein receptor-related protein 2;LRP2	9.6516
6.140096379	0.021296121	Low-density lipoprotein receptor-related protein 5-like protein;LRP5L	1.45528
6.128036496	0.034290655	Coiled-coil domain-containing protein 40;CCDC40	9.55E-145
6.074112707	0.02373428	Cilia- and flagella-associated protein 45;CFAP45	4.7E-57
6.037449072	0.020767261	Dynein assembly factor 1, axonemal;DNAAF1	2.2E-84
5.693642304	0.027202649	Forkhead box protein J1;FOXJ1	5.18E-36
5.466338354	0.012384784	Coiled-coil domain-containing protein 103;CCDC103	1.32E-15
5.395582726	0.024207826	Transmembrane protein 107;TMEM107	9.4E-17
5.337766651	0.031895306	Protein fantom;RPGRIP1L	8.75E-162
5.261625929	0.030427328	Low-density lipoprotein receptor-related protein 4;LRP4	2.64317
5.187814436	0.006367972	Cytoplasmic dynein 2 light intermediate chain 1;DYNC2LI1	8.1E-73
5.152784143	0.016016252	ADP-ribosylation factor-like protein 6;ARL6	1.38E-69
5.149220171	0.001002666	Cadherin EGF LAG seven-pass G-type receptor 2;CELSR2	1.17744
5.091208494	0.024291933	Intraflagellar transport protein 52 homolog;IFT52	3.55779
5.031722355	0.017517025	Forkhead box protein G1;FOXG1	3.63E-53
5.017456886	0.049878081	Dynein heavy chain 5, axonemal;DNAH5	0
