# Supplementary material for: Regeneration of Planarian Auricles and Reestablishment of Chemotactic Ability
Source: Front Cell Dev Biol. 2021 Nov 26;9:777951. doi: 10.3389/fcell.2021.777951 (PMC8662385; doi:10.3389/fcell.2021.777951)
Supplement: Supplementary file 9 [file DataSheet1.PDF]

SUPPLEMENTARY FILE LEGENDS

Supplementary File S1. *Girardia dorotocephala* MA-C2 reference transcriptome (v.2021). FASTA file containing 268,178 supercontig sequences generated from *de novo* assembly of Illumina RNAseq reads from *G. dorotocephala* clonal line MA-C2 intact planarians and auricle fragments. File available for download at https://corescholar.libraries.wright.edu/biology/802/

Supplementary File S2. Analysis of differential gene expression in *G. dorotocephala* auricles. Exel (.xlsx file) containing the IDs of 268,178 reference transcriptome supercontigs (1st column) as well as matching accession number, description, and E-value of top BLASTX human protein sequence matches (2nd, 3rd, and 4th column). Fold-change enrichment between average transcripts per million (TPM) mapped from auricle reads (14th column) as compared to average TPM from body reads (13th column) is shown (5th column). *p*-value calculated using Student's *t*-test analysis of TPM counts from three independent sets of body (7th-9th columns) and auricle (10th-12th columns) reads is included as statistical assessment of differential gene expression (6th column). Sum of total TPMs from all samples mapped to each contig are included (15th column). File available for download at https://github.com/josephryan/Almazan_et_al_auricles_regen/

Supplementary Table S1: Enriched GO groups amongst homologs of auricle-enriched transcripts. Gene Ontology (GO) biological process groups (left column) significantly enriched amongst human homologs of genes with $\geq$ 5-fold higher expression in auricles than in whole planarian bodies. Fold-enrichment (2nd column) of GO group members as compared to expected from representation in the human genome, *p*-value from Fisher's exact test (3rd column) and False Discovery Rate (FDR; 4th column) are shown. Applied GO Ontology database DOI: 10.5281/zenodo.5228828 Released 2021-08-18

Supplementary Table S2. *G. dorotocephala* auricle-enriched orthologs of genes with decreased expression upon extended *SoxB1* RNAi in *S. mediterranea.* Table listing IDs of 31 *G. dorotocephala* supercontigs (1st column) with more than 5-fold enriched expression in auricles and corresponding *S. mediterranea* orthologous sequences (2nd column). Description, accession number, and E-value (3rd, 4th, and 5th column) of top BLASTX human protein sequence matches to *G. dorotocephala* sequences, as well as fold-change enrichment between average transcripts per million (TPM) mapped from auricle reads as compared to average TPM from body reads is shown (6th column). *p*-value calculated using Student's *t*-test analysis of TPM counts from three independent sets of body and auricle reads is included as statistical assessment of differential gene expression in *G. dorotocephala* auricles (7th column).

Supplementary Table S3. Supplementary Table S2. Homologs of genes with enriched expression in auricles belonging to GO: "pattern specification process". Fold- (first column) and statistical significance (according to Student's t-test; *p*-value; second column) of enriched representation of mapped auricle reads, relative to whole-body mapped reads, whose top match homolog belongs to the "pattern specification process" GO group. Gene name and symbol (third column), as well as BLASTX E-value (fourth column), of top human protein sequence matches are included. Genes with poor BLASTX E-value ($\geq$ 0.1) are indicated in gray font.

Figure S1. Principal component analysis of mapped RNAseq reads from *G. dorotocephala* auricles and bodies. Principal component analysis of Illumina reads from auricles and intact or auricle-less bodies from clonal (cl) and non-clonal (w) lines of *G. dorotocephala* mapped to the reference transcriptome show segregation of auricle and body reads along the fists principal component (PC1) and separation of reads from clonal and non-clonal lines along the second principal component (PC2).

Figure S2. Planarians foraging ability is challenged by vertical distance of chemoattractant. (A) Dimensions of chemotaxis chambers utilized to assess the vertical range of chemoreception. **(B)** Difficulties in *G. dorotocephala* feeding success are observed when the distance of the chemoattractant is increased from 5 (blue line) to 10 cm (red line) from the bottom of the chamber, and almost complete failure to reach the food is observed when positioned 45 cm from the bottom of the chamber (green line). Note that *G. dorotocephala* normally achieves 80-90% feeding success within 30 minutes when positioned at a similar distance horizontally away from the chemoattractant (See Figure 3 and Figure 4 in main text).

Figure S3. Auricles do not appear to contribute to the overall motility or light avoiding behavior of *G. dorotocephala*. (A-B) Planarians tested for 1-day post-auricle amputation display overall normal chemotactic response. Arena used in negative phototaxis assays of *G. dorotocephala* has a 7 cm radius and a light is positioned over the area labeled I (A). Luminosity decreases from area I to IV, and the time each planarian spend in each section of the arena is measured for three minutes. Average percent time spent in each section by of intact (gray bars) and auricle amputees (white bars) from each of two independent biological replicates ($n \geq 6$ per group per replicate) is plotted in (B), showing that both groups preferred the area furthest from the light source. A slight but significant difference in the time spent in section I (Student's *t*-test, *p*-value ≤ 0.05; asterisk) may be due to defects in response to heat emitted by the light source due to potential loss of thermo-sensory cells positioned in the area of the auricle as observed in other planarian species (Inoue et al., 2014; Arenas et al., 2017). **(C-E)** Significant differences were not observed between the two groups in average speed (C) or initial movements of planarians when dropped into a test arena without a light gradient (D and E). **(F and G)** Contribution of auricles to negative chemotaxis was tested in an arena (F) of dimensions as in (A) with agarose containing turmeric positioned in quadrant "d" and planarians in quadrant "a" at the start of the assay. Measurement of time spent by intact and auricle amputees one day post-amputation (1DPA) in quadrants "a", "b" and "c", or "d" failed to reveal significant difference (n.s.) in avoidance behavior between the two groups.

Figure S4. Quantitative analysis of mitotic and ciliated cell distribution during the first 2 days post-auricle amputation. Maximum intensity projection of confocal z-stack images from intact (A) and auricle amputees 6 hrs. post-amputation (6 HPA; B), 12 HPA (C), 1 day post-amputation (1 DPA; D and G) and 2 DPA (E and H), illustrate distribution of M-phase neoblasts stained with phospho-Histone H3 (PH3; orange) and ciliated cells stained with anti-acetylated tubulin (AcTub; green). DAPI staining of cell nuclei reveals the general position of cells. Graphs show quantification of the average number of PH3+ cells positioned anterior to the eyes (F) and the area of aggregated ciliated cells (dashed lines) at the position of the auricle (I). White

asterisks (*) in (A-E) mark position of eye. Black asterisks in (F) indicates statistical significance (Student's *t*-test, *p*-value ≤ 0.05; asterisk). Scale = 0.1 mm.

Figure S5. Conserved sequences are enriched in candidate auricle genes when compared to entire reference transcriptome. Pie charts displaying percent fraction of contigs matched to human protein sequences with an E-value < 10E-2 (pink) in BLASTX analyses of the entire reference transcriptome (left) and of sequences with ≥ 5-fold (*p*-value ≤ 0.05) enriched abundance in RNAseq analysis of auricles (right).

Figure S6. Position of genes of interest in auricle RNAseq analysis. Log plot of average transcripts per million (TPM) calculated from Illumina reads of RNA extracted from *G. dorotocephala* auricles (*y* axis) and bodies (*x* axis) mapped to reference transcriptome contigs. Contigs with (Student's *t*-test, *p*-val < 0.05; blue) and without (gray) statistically significant differences in relative gene expression are marked. Contigs with statistically significant difference in gene expression and ≥ 5-fold enriched abundance in reads of auricles are shown in red. The position of specific genes of interest based on previous studies are enlarged, labeled, and highlighted in yellow. The genes include Piwi homologs, which were significantly lower in auricle reads as compared to reads from the body, as would be expected of neoblasts markers. *GdCPEB2*, which is a gene that is broadly expressed in the planarian central nervous system (CNS; Rouhana et al., 2018; Almazan et al., 2018) and not enriched in reads of the auricle (1.05-fold; *p*-value = 0.86), suggesting that neuronal tissue is not more abundant in auricles than in the animal as a whole. The *G. dorotocephala* ortholog of *Smed-fli-1*, a gene expressed in brain branches and required for food chemotaxis in *S. mediterranea* (Roberts-Galbraith et al., 2016), was slightly enriched in reads of the auricles (1.52-fold; *p*-value < 0.05). The *G. dorotocephala* ortholog of *Dj-fgf*, a gene specifically expressed in the auricles and pharynx of *D. japonica* (Auwal et al., 2020) was also slightly but not significantly enriched (1.36-fold; *p*-value > 0.05). Also highlighted are orthologs of *Smed-cav1* and *Smed-cng-1*, which are genes highly expressed in *S. mediterranea* sensory neurons (Ross et al., 2018) and subepithelial cells at the distal end of brain branches (Roberts-Galbraith et al., 2016), respectively (6.8-fold and 13.4-fold, respectively; *p*-value < 0.05). Other genes of interest not highlighted in this figure include homologs of a receptor for retinol uptake (STRA6), which was enriched 20-fold; genes with some degree of detectable sequence conservation with human olfactory receptors (O5H14, OE1L6, OR4X2, OR2F1, O51G2) that ranged between 5-fold and 16-fold enrichment in auricles; as well as homologs of genes associated with human neurological disorders such as Fetal Alcohol Syndrome (FEZF2; 18-fold), Spinocerebellar Ataxia (SNX13; 11.6-fold), Retinis Pigmentosa (NR2E1; 36- and 28-fold), and Niemann-Pick Disease (NEGR1; 10-fold). The entire dataset is available in Supplementary File S2.
